# Ethnic and socioeconomic inequalities in stroke risk factors and primary prevention: the South London Stroke Register cohort study 1995–2024

**DOI:** 10.1016/j.eclinm.2026.104061

**Published:** 2026-07-09

**Authors:** Eva S. Emmett, Camila Pantoja-Ruiz, Evelyn Lim, Amal R. Khanolkar, Ajay Bhalla, Charles D.A. Wolfe, Matthew D.L. O'Connell, Iain J. Marshall

**Affiliations:** a Department of Population Health Sciences, King's College London, London, United Kingdom; b NIHR Applied Research Collaborative South London, London, United Kingdom; c Department of Ageing and Health, Guy's and St Thomas' NHS Foundation Trust, London, United Kingdom

**Keywords:** Stroke, Vascular risk factors, Health inequality, Ethnicity, Socioeconomic status, Primary prevention

## Abstract

**Background:**

Ethnic minority and lower socioeconomic groups experience higher stroke incidence. This study investigates long-term trends of inequalities in vascular risk factor prevalence and management, and whether socioeconomic inequalities explain ethnic inequalities.

**Methods:**

8515 participants of the population-based South London Stroke Register cohort (1995–2024) were stratified by ethnicity (62.4% White, 15.5% Black Caribbean, 13.3% Black African), occupation (60.7% routine/manual), education (44.6% lower education), and stroke year (1995–2004, 2005–2014, 2015–2024). The impact of ethnicity, occupation and education on risk factor diagnoses and prescribed treatments was estimated using Poisson regression models.

**Findings:**

12.0% of strokes in Black African participants (median age 59.0 years) occurred without pre-stroke risk factor diagnosis (White participants: 6.3%, median age 74.1 years). Black and lower socioeconomic groups had higher rates of hypertension (adjusted prevalence ratio: Black Caribbean vs White: 1.29 [95% CI: 1.20–1.34], Black African: 1.47 [1.40–1.53], manual/routine vs non-routine/non-manual occupation: 1.09 [1.05–1.13], lower vs higher education: 1.06 [1.02–1.11]) and diabetes (Black Caribbean: 2.23 [2.04–2.43], Black African: 1.92 [1.73–2.13], manual/routine: 1.23 [1.13–1.34], lower education: 1.21 [1.09–1.37]) but lower rates of atrial fibrillation (Black Caribbean: 0.57 [0.48–0.68], Black African: 0.66 [0.54–0.82], lower education: 0.78 [0.68–0.91]). Over time (1995–2024), ethnic and socioeconomic inequalities widened for diabetes and hypertension but narrowed for atrial fibrillation. 36% of strokes occurred in people with diagnosed, but untreated risk factors. Treatment rates were not associated with occupation or education, but Black participants had higher treatment rates for hypertension and diabetes. Adjusting for occupation or education had negligible impact on associations between ethnicity and risk factor diagnosis or treatment.

**Interpretation:**

Prevalence of pre-stroke hypertension and diabetes is higher and increasing faster in ethnic minority and lower socioeconomic groups. Together with Black people's younger stroke age and higher likelihood of having stroke without prior risk factor diagnosis, these findings call for targeted and earlier primary prevention efforts.

**Funding:**

This project is funded by the National Institute for Health and Care Research (NIHR) under its Programme Grants for Applied Research (NIHR202339).

**Research in context**

**Evidence before this study**

We searched PubMed for publications from January 2005 to June 2025 using a search strategy that combined terms for “stroke”, “ethnic”, “socioeconomic”, “inequality”, “vascular risk factors”, and “primary prevention”. Previous studies showed that ethnic minority and lower socioeconomic groups have higher vascular risk factor burden, which significantly contribute to inequalities in stroke incidence. However, long-term trends of inequalities in vascular risk factor burden are poorly characterised and the extent to which ethnic inequalities are explained by socioeconomic inequalities remains unclear.

**Added value of this study**

Using data from a population-based prospective cohort study with continuous recruitment covering 30 years, this study found widening ethnic and socioeconomic inequalities in pre-stroke diabetes and hypertension, while inequalities in atrial fibrillation have narrowed. Socioeconomic disadvantage and ethnic minority status independently affect pre-stroke risk factor profiles. Pre-stroke risk factor detection and treatment rates remain suboptimal across all groups.

**Implications of all the available evidence**

Vascular risk factor detection and control, particularly of hypertension and diabetes in ethnic minority and lower socioeconomic groups, are critical to reducing widening stroke incidence inequalities. The current start of vascular health checks in the UK at age 40 might miss primary prevention opportunities in high-risk groups with their often-younger age at stroke. Suboptimal risk factor detection and treatment rates highlight a need for universal as well as targeted improvement strategies.

## Introduction

Stroke remains a major cause of morbidity and mortality worldwide and in the UK; yet ∼90% of strokes are associated with ten modifiable vascular risk factors and are therefore potentially preventable.^1^ Despite the availability of evidence-based guidelines and pay-for-performance policies in many countries, including the UK,^2^ risk factor control remains inadequate, due to shortcomings in detection, prescription, medication adherence, and/or monitoring.^3^

Ethnic inequalities in stroke incidence persist, significantly driven by inequalities in risk factor prevalence and control,^3^^,^^4^ and with strokes occurring at younger ages in ethnic minority groups.^5^ In previous studies, Black ethnic groups had higher rates of hypertension and lower treatment and control rates compared to their White counterparts, even though suboptimal control confers a three times higher stroke risk.^6^ An American study described a steeper rise of diabetes in Black compared to White stroke patients.^7^ Conversely, lower atrial fibrillation (AF) prevalence has been reported in Black ethnic groups, despite a greater burden of AF risk factors, referred to as the “AF paradox”^8^; and anticoagulation remains widely under-used, especially in ethnic minority groups.^9^

Higher risk factor prevalence and lower treatment and control rates have also been reported for lower socioeconomic groups.^10^^,^^11^ Due to the intersectionality between ethnicity and socioeconomic status, these characteristics may compound to increase an individual's risk factor burden and subsequent stroke risk.^12^

While ethnic and socioeconomic inequalities in vascular risk factors have been reported, long-term trends in population-based stroke cohorts and the extent to which socioeconomic inequalities drive ethnic inequalities remain understudied.^11^^,^^12^ The South London Stroke Register (SLSR) benefits from a large cohort of significant ethnic and socioeconomic diversity covering three decades. This allows detailed analyses of long-term trends in ethnic and socioeconomic inequalities and their mutual impact. Compared to American studies, this study's UK setting with its universal, tax-funded healthcare provision adds a different lens to studying health inequalities. Identifying predominant risk factors and suboptimal treatment patterns in specific groups can inform more targeted approaches of vascular health checks, lifestyle campaigns, and risk factor control. Long-term trends in risk factor diagnoses contribute to evaluating past prevention and detection efforts, estimating future trends, and informing future policies.

Using SLSR data, we investigated inequalities in pre-stroke risk factor diagnoses and treatments between ethnic and socioeconomic groups, their mutual impact, and trends over 30 years.

## Methods

The SLSR is a population-based cohort study of adults with first stroke since 1995 in a defined area of inner-city London, UK. The study area's population increased from 310,028 in 2001 (63% White, 15% Black African, 9% Black Caribbean, 13% other ethnic groups, hereafter “Other”) to 398,555 in 2021 (52% White, 15% Black African, 7% Black Caribbean, 26% Other).^13^

The methods of the SLSR have been described previously^14^ and are summarised here. Multiple overlapping notification sources were used to enhance case ascertainment, including hospital admissions, outpatient clinics, radiology reports, A&E records, and general practitioners. Capture-recapture models estimated 88% completeness of case ascertainment.^15^ Stroke diagnosis was verified by senior study clinicians using the World Health Organization definition of stroke ICD-10 until March 2022 and ICD-11 thereafter.

### Ethics

The study has approval from the NHS Health Research Authority (22/WA/0027) and previously from the ethics committees of Guy's and St Thomas' Hospital, King's College Hospital, Queen's Square, and Westminster Hospital. Informed consent or consultee advice was obtained for all participants. The study adhered to the principles of the Declaration of Helsinki.

### Exposure

Ethnicity was self-reported and stratified as per the UK census^13^ and most common groups in this cohort into White, Black Caribbean, Black African and Other (other Black groups/Asian/mixed/other).

Socioeconomic status (SES) was estimated using the individual-level indicators occupation and education. Occupation was defined as current or most recent occupation if currently not working/retired or spouse's/father's occupation for stay-at-home spouses/students. Until 2007, occupations were categorised into “manual” (skilled/un-skilled) or “non-manual” (professional/managerial/intermediate/non-manual skilled) according to UK General Register Office occupational codes (Categories I–III/N and III/M–V respectively). Since 2008, the three-class version of the National Statistics Socioeconomic classification (NS-SEC) was used (managerial/administrative/professional; intermediate; or routine/manual).^16^ To create a single binary “occupation” variable covering the entire study period, the two higher NS-SEC categories were further combined into “non-routine/non-manual” and both variables subsequently merged.

Education was recorded since 2004 and dichotomised into “lower education” (no formal education, primary, lower secondary education) and “higher education” (upper secondary, post-secondary non-tertiary, tertiary education).

### Outcomes

Pre-stroke risk factor diagnoses were collected as binary variables from hospital/GP records, including hypertension, hypercholesterolaemia (collected from 1997), diabetes mellitus, myocardial infarction, atrial fibrillation (AF), and body mass index (BMI ≥25 categorised as overweight/obese, from 2001). Smoking (current or ex-smoker/never) was obtained from participant interviews. AF newly diagnosed at stroke was collected since 2002.

Pre-stroke risk factor treatments were based on prescribed treatments, collected from hospital/GP records. They include antihypertensives, antiplatelets, anticoagulants, and cholesterol-lowering and diabetes medication. Treatment rates are expressed as percentages of participants diagnosed with the relevant risk factor, e.g. antihypertensive treatment in those diagnosed with hypertension. Additionally, anticoagulation rates of participants with AF and CHA_2_DS_2_-VASc score ≥2 in men and ≥3 in women (“high-risk AF”) were calculated. The outcome variable “at least one untreated risk factor” was created as a binary variable distinguishing between those participants who had appropriate treatment for all their diagnosed vascular risk factor (out of hypertension, diabetes, hypercholesterolaemia, AF, myocardial infarction, and TIA) and those that did not receive treatment for one or more of these risk factors.

Stroke subtype was stratified into ischaemic and haemorrhagic (primary intracerebral/subarachnoid). Since 1999 the Trial of Org 10172 in Acute Stroke Treatment (TOAST) classification was collected.

This study was reported using the Strengthening the Reporting of Observational Studies in Epidemiology (STROBE) guidelines.

### Statistical analyses

Participants with first stroke between 1995 and 2024 were stratified by ethnicity, occupation, and education and into three 10-year cohorts by stroke year (1995–2004, 2005–2014, 2015–2024). Stroke year was categorised into cohorts for descriptive purposes and to allow the evaluation of non-linear trends over time. These specific 10-year cohorts were chosen for sample size considerations but also as meaningful from a clinical perspective (introduction of the UK Quality and Outcomes Framework^2^ in 2004 aimed at improving primary care quality and 2014 update of the NICE AF clinical guideline).^17^ Categorical variables were summarised as count (percentage). The continuous variable age showed a skewed distribution (on visual inspection of data distribution) and was therefore summarised as median (interquartile range [IQR]). Bivariate analyses were performed using chi^2^-test, and Wilcoxon rank-sum and Kruskal–Wallis test as appropriate. P-values for trend over time were calculated using the Cochran–Armitage test for categorical variables and Cuzick test for medians.

Multivariable Poisson regression models with robust standard errors were used to estimate adjusted prevalence ratios (aPR) of pre-stroke risk factor diagnoses and treatment rates among ethnic minority versus White participants, those with routine/manual versus non-routine/non-manual occupations, and those with lower versus higher education. aPRs were estimated separately for ethnicity (model 1), occupation (model 2), and education (model 3). Models were run for the whole study population and for each cohort. To investigate whether SES explained ethnic inequalities, a second set of models were mutually adjusted for either ethnicity and occupation (model 4), or ethnicity and education (model 5). In further models, interaction terms were included between ethnicity and occupation (model 6), or ethnicity and education (model 7) to examine whether the effect of ethnicity varied by SES, and vice versa. Finally, to test whether aPRs between groups changed over time, we included interaction terms between ethnicity and cohort (model 8) or occupation and cohort (model 9); interactions with education were not included as only recorded from 2004. All models were additionally adjusted for age, sex, and year of stroke, including within cohorts.

SES indicators had significant proportions of missing values (occupation: 23.3%, education: 27.3% from 2004). Data completeness was over 95% for all outcome variables, apart from hypercholesterolaemia (7.9% missing from 1998/collection start), cholesterol lowering treatment (8.5% missing), smoking (9.9%), and BMI (34.0% from 2001; Table S1/Supplement).

Baseline characteristics of those with and without missing indicator were compared to assess for potential bias (Table S2/Supplement). Missingness was significantly associated with several of the included observed variables, hence a MAR assumption is plausible. We have additionally carried out Little's MCAR test which provided evidence that data are not MCAR (p < 0.001).

To further test robustness of the results, multiple imputations with chained equations were conducted. As education was only recorded since mid-2004, two imputed datasets were created, one including the whole study population but without the variable education and another including education covering 2005–2024. Based on the percentage of incomplete observations, 50 datasets were imputed respectively. Parameter estimates were combined using Rubin's rules. Poisson regression models using imputed datasets were used to estimate aPRs of pre-stroke risk factor diagnoses and treatment rates (Table S3/Supplement) but were not meaningfully different from complete case analyses. Complete case analyses are therefore presented as main analyses. When occupation or education served as covariates, missing values were included as separate categories.

All tests were 2-tailed, and P < 0.05 was considered statistically significant. Analyses were performed using Stata 18.0 (StataCorp, Texas/USA).

### Role of the funding source

The funders had no role in the study design; in the collection, analysis, and interpretation of data; in the writing of the report; and in the decision to submit the article for publication.

## Results

Between 1995 and 2024, a total of 8643 participants were registered. 128 participants had no ethnicity, education, and occupation recorded and were excluded (Table S4/Supplement), leaving 8515 participants for analysis. 5255 (62%) participants self-reported as White, 1303 (16%) as Black Caribbean, and 1122 (13%) as Black African (Table 1). 3964 (61%) reported routine/manual occupations and 1929 (45%) lower education. Black African participants were younger at first stroke (median age: 59.0) than Black Caribbean (69.4) and White participants (74.1, Table 2) with age disparities declining over time (Table S5A/Supplement). Black Caribbean participants had the highest proportion of routine/manual occupation, while Black African participants had the highest proportion of higher education, even after adjusting for age, sex, and stroke year (Table S6/Supplement).

**Table 1:** Characteristics of the study population (N = 8515, 1995–2024), overall and by cohorts.

	Overall	1995–2004	2005–2014	2015–2024	P trend
	N = 8515	N = 2907	N = 2697	N = 2911	
Age, years (median, IQR)	70.5 (58.9–80.5)	72.8 (62.7–81.1)	71.0 (58.2–87.1)	67.4 (56.7–79.3)	<0.001
Sex, female	4052 (47.6%)	1459 (50.2%)	1286 (47.7%)	1307 (44.9%)	<0.001
Ethnicity					
White	5255 (62.4%)	2155 (75.4%)	1693 (63.3%)	1407 (48.8%)	<0.001
Black Caribbean	1303 (15.5%)	352 (12.3%)	435 (16.3%)	516 (17.9%)	<0.001
Black African	1122 (13.3%)	182 (6.4%)	318 (11.9%)	622 (21.6%)	<0.001
Other	740 (8.8%)	170 (5.9%)	230 (8.6%)	340 (11.8%)	<0.001
Occupation					
Non-routine/non-manual	2569 (39.3%)	757 (31.5%)	819 (40.4%)	993 (47.2%)	<0.001
Routine/manual	3964 (60.7%)	1643 (68.5%)	1208 (59.6%)	1113 (52.8%)	<0.001
Education^a^					
Higher education	2394 (55.4%)	44 (24.2%)	1054 (52.4%)	1296 (60.9%)	<0.001
Lower education	1929 (44.6%)	138 (75.8%)	959 (47.6%)	832 (39.1%)	<0.001
Number of VRFs					
None	599 (7.3%)	140 (5.1%)	187 (7.2%)	272 (9.6%)	<0.001
1 or 2	4684 (57.4%)	1801 (65.8%)	1485 (57.2%)	1398 (49.3%)	<0.001
≥3	2882 (35.3%)	796 (29.1%)	922 (35.5%)	1164 (41.1%)	<0.001
≥1 untreated VRF	2711 (35.8%)	994 (38.3%)	799 (33.2%)	918 (35.8%)	0.065
Hypertension	5390 (66.0%)	1744 (64.4%)	1751 (66.2%)	1895 (67.4%)	0.021
Antihypertensive treatment∗	3906 (74.2%)	1050 (63.4%)	1299 (75.1%)	1557 (83.0%)	<0.001
Diabetes mellitus	2187 (26.5%)	589 (21.7%)	606 (22.8%)	992 (34.6%)	<0.001
Diabetes treatment∗	1637 (76.2%)	474 (83.7%)	489 (81.2%)	674 (68.8%)	<0.001
Atrial fibrillation	1332 (16.4%)	473 (17.4%)	419 (16.0%)	440 (15.9%)	0.136
Newly diagnosed atrial fibrillation	302 (6.1%)	45 (7.7%)	150 (7.2%)	107 (4.8%)	0.001
Anticoagulants if AF∗	385 (29.6%)	69 (15.4%)	93 (22.4%)	223 (51.1%)	<0.001
Anticoagulants if high-risk AF∗	318 (34.4%)	31 (17.6%)	86 (23.6%)	201 (52.2%)	<0.001
Antiplatelets if AF∗	505 (38.8%)	172 (38.3%)	212 (51.0%)	121 (27.8%)	0.002
Hypercholesterolaemia	2219 (31.7%)	228 (14.5%)	850 (32.4%)	1141 (40.8%)	<0.001
Cholesterol-lowering treatment∗	1659 (76.4%)	138 (68.3%)	617 (73.5%)	904 (80.1%)	<0.001
Myocardial infarction	911 (11.2%)	316 (11.6%)	232 (8.9%)	363 (13.1%)	0.082
TIA	856 (10.7%)	354 (13.3%)	223 (8.6%)	279 (10.1%)	<0.001
Antithrombotics if TIA or MI∗	1016 (65.3%)	323 (55.9%)	307 (73.1%)	386 (69.3%)	<0.001
Smoking, current or ex	4369 (57.0%)	1673 (62.3%)	1440 (59.6%)	1256 (49.1%)	<0.001
Pre-stroke BMI ≥ 25^b^	2501 (56.3%)	289 (50.1%)	923 (57.0%)	1289 (57.5%)	0.008
Stroke type					
Haemorrhagic stroke	1586 (19.0%)	562 (20.5%)	433 (16.1%)	591 (20.3%)	0.936
Ischaemic stroke	6752 (81.0%)	2182 (79.5%)	2252 (83.9%)	2318 (79.7%)	
TOAST^c^					
LAA	562 (9.0%)	100 (7.8%)	246 (10.8%)	216 (8.1%)	0.638
CE	1306 (21.0%)	294 (23.0%)	480 (21.0%)	532 (20.0%)	0.040
SVO	1302 (20.9%)	300 (23.4%)	478 (20.9%)	524 (19.7%)	0.010
OTH/UND	1811 (29.1%)	305 (23.8%)	681 (29.8%)	825 (31.1%)	<0.001
PICH	926 (14.9%)	195 (15.2%)	301 (13.2%)	430 (16.2%)	0.155
SAH	314 (5.0%)	87 (6.8%)	99 (4.3%)	128 (4.8%)	0.0317

Summary statistics are count (%); Percentages refer to those with known value as denominator; when indicated (∗) referring to those with relevant VRF diagnosis; p-value for trend across cohorts was calculated using Cochran–Armitage test of trend for categorical variables.

**Abbreviations:** VRFs: Vascular risk factors, BMI: body mass index, TOAST classification: Trial of Org 10172 in Acute Stroke Treatment classification, LAA: large artery atherosclerosis, CE: cardioembolic, SVO: small vessel occlusion, Oth/UND: other or undefined ischaemic stroke, PICH: primary intracerebral haemorrhage, SAH: subarachnoid haemorrhage.

a Education recorded since 2004.

b “BMI” recorded since 2001.

c “TOAST classification” collected since 1999.

**Table 2:** Characteristics of the study population, stratified by ethnicity.

	White	Black Caribbean	Black African	Other	p-value
	N = 5255	N = 1303	N = 1122	N = 740	
Age, years (median IQR)	74.1 (63.0–82.5)	69.4 (59.6–78.7)	59.0 (50.4–69.0)	65.5 (54.2–75.6)	<0.001
Sex, female	2556 (48.6%)	628 (48.2%)	492 (43.9%)	332 (44.9%)	0.012
Occupation					<0.001
Non-routine/non-manual	1634 (40.6%)	275 (27.4%)	397 (44.0%)	220 (42.8%)	
Routine/manual	2390 (59.4%)	727 (72.6%)	505 (56.0%)	294 (57.2%)	
Education^a^					<0.001
Higher education	1237 (52.3%)	372 (50.7%)	521 (67.5%)	241 (57.9%)	
Lower education	1128 (47.7%)	362 (49.3%)	251 (32.5%)	175 (42.1%)	
Number of VRFs					<0.001
None	318 (6.3%)	75 (5.9%)	130 (12.0%)	76 (10.8%)	
1 or 2	2953 (58.8%)	687 (54.0%)	625 (57.8%)	371 (52.6%)	
≥3	1754 (34.9%)	511 (40.1%)	327 (30.2%)	258 (36.6%)	
≥1 untreated VRF	1847 (39.2%)	448 (37.4%)	331 (34.8%)	242 (38.5%)	0.066

Summary statistics are count (%); Percentages refer to those with known value as denominator.

**Abbreviation:** VRFs: Vascular risk factors.

a Education recorded since 2004.

Overall, the proportion of participants with three or more vascular risk factors increased from 29% in 1995–2004 to 41% in 2015–2024, while the proportion of strokes occurring without pre-stroke risk factor diagnosis also increased from 5% to 10% (Table 1). Prevalence increased most strongly for diabetes (22%–35%) and hypercholesterolaemia (15%–41%) but also for hypertension and overweight/obesity, while smoking and TIA declined.

### Inequalities in overall risk factor burden and trends over time

**Fig. 1:**
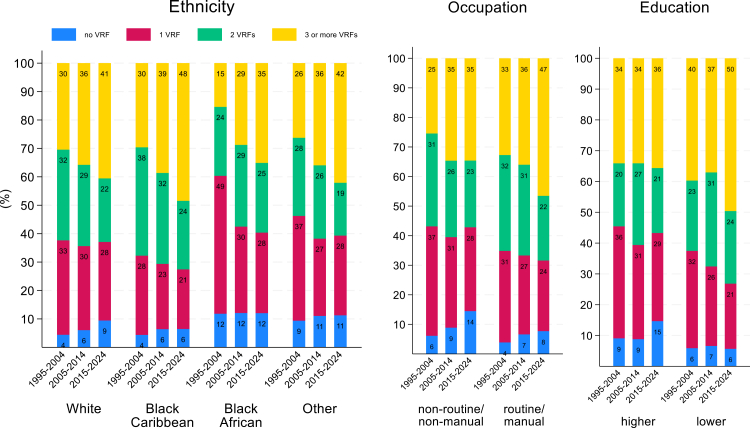
**Trends in the proportion of participants with none, one, two, and three or more pre-stroke vascular risk factors, stratified by ethnicity, occupation, and education, and year of stroke. p-values for trends across cohorts,** calculated using Cochran–Armitage test of trend: **No VRF:** White <0.001, Black Caribbean 0.235, Black African 0.962, Other 0.554; non-routine/non-manual <0.001; routine/manual <0.001; higher education <0.001, lower education 0.580; **One VRF**: White <0.001, Black Caribbean 0.023, Black African <0.001, Other 0.078; non-routine/non-manual <0.001; routine/manual <0.001; higher education 0.183, lower education 0.002; **Two VRFs**: White <0.001, Black Caribbean <0.001, Black African 0.671, Other 0.016; non-routine/non-manual <0.001; routine/manual <0.001; higher education 0.009, lower education 0.058; **Three or more VRFs**: White <0.001, Black Caribbean <0.001, Black African <0.001, Other <0.001; non-routine/non-manual <0.001; routine/manual <0.001; higher education 0.453, lower education <0.001.

The proportion of strokes occurring without prior risk factor diagnosis was higher for Black African participants (12%) than Black Caribbean or White participants (both 6%, Table 2). Three or more vascular risk factor diagnoses were more common among Black Caribbean participants (40%) than White (35%) or Black African participants (30%). Between 1995–2004 and 2015–2024, this proportion increased more strongly in Black Caribbean (30%–48%) and Black African (15%–35%) than White participants (30%–41%, Fig. 1). Similarly, lower-SES participants had higher proportions with three of more risk factors than higher-SES participants (38% vs 32% (by occupation) and 43% vs 35% (by education), Table 3), again increasing more strongly in lower SES-participants over time (Fig. 1).

**Table 3:** Characteristics of the study population, stratified by occupation and education.

	Occupation			Education		
	Non-routine/non-manual	Routine/manual	p-value	Higher education	Lower education	p-value
	N = 2569	N = 3964		N = 2394	N = 1929	
Age, years (median, IQR)	69.0 (56.4–79.6)	69.8 (59.4–79.4)	<0.001	63.5 (52.9–74.9)	72.1 (60.9–81.1)	<0.001
Sex, female	1248 (48.6%)	1656 (41.8%)	<0.001	1040 (43.4%)	855 (44.3%)	0.56
Ethnicity			<0.001			<0.001
White	1616 (64.0%)	2338 (59.7%)		1237 (52.2%)	1128 (58.9%)	
Black Caribbean	275 (10.9%)	714 (18.2%)		372 (15.7%)	362 (18.9%)	
Black African	397 (15.7%)	502 (12.8%)		521 (22.0%)	251 (13.1%)	
Other	238 (9.4%)	362 (9.2%)		241 (10.2%)	175 (9.1%)	
Number of VRFs			<0.001			<0.001
No VRF	257 (10.3%)	225 (5.8%)		282 (12.0%)	118 (6.2%)	
1 or 2 VRFs	1441 (57.7%)	2187 (56.5%)		1249 (53.1%)	981 (51.2%)	
≥3 VRFs	798 (32.0%)	1456 (37.6%)		821 (34.9%)	817 (42.6%)	
≥1 untreated VRF	729 (32.6%)	1288 (35.4%)	0.028	672 (32.5%)	568 (31.6%)	0.562

Summary statistics are count (%); Percentages refer to those with known value as denominator.

**Abbreviation:** VRFs: Vascular risk factors.

### Inequalities in individual risk factor prevalence

**Fig. 2:**
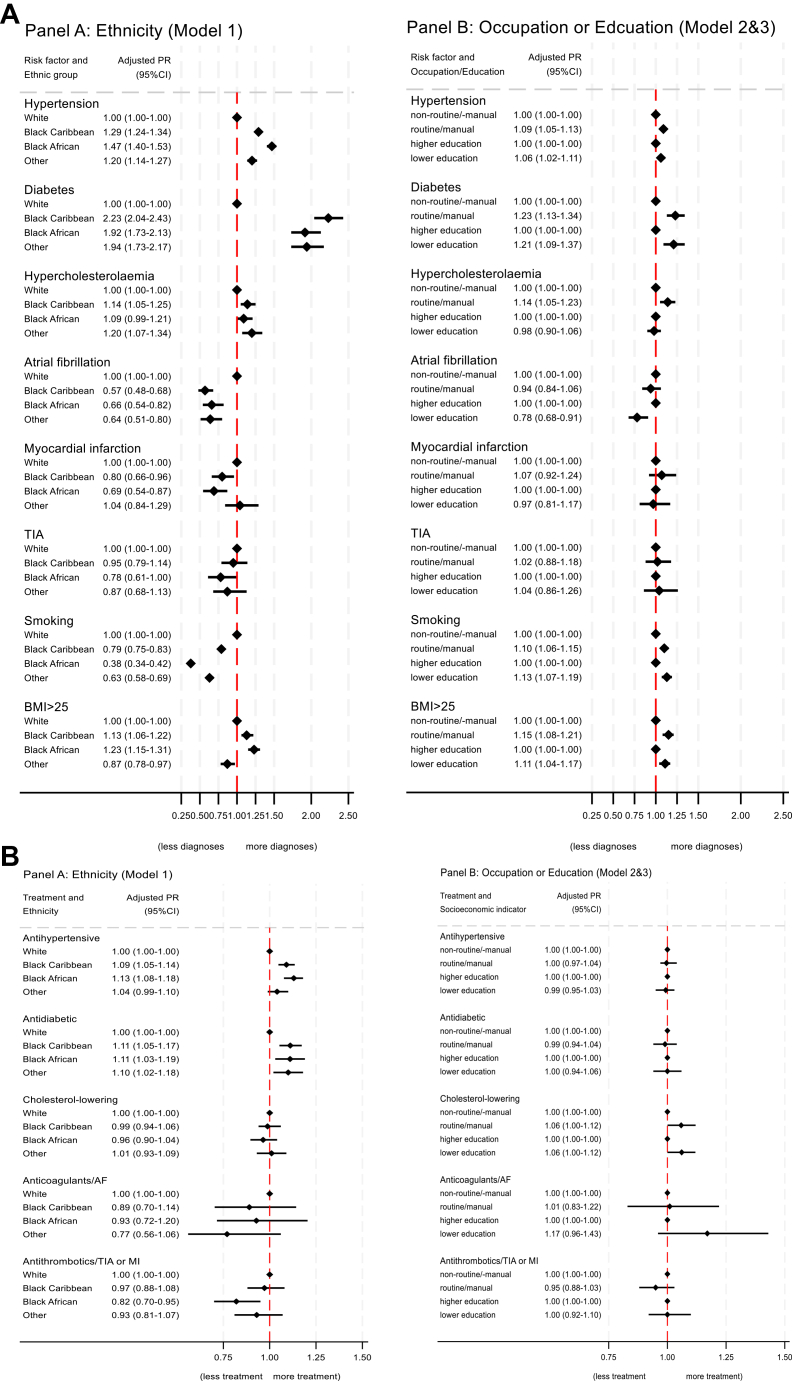
**A: Adjusted prevalence ratios of pre-stroke vascular risk factor diagnoses between ethnic and socioeconomic groups. B: Adjusted prevalence ratio of pre-stroke treatments in those diagnosed with respective risk factor between ethnic and socioeconomic groups. Model 1:** ethnic minority groups versus White group (reference), **Model 2:** individuals with routine/manual versus non-routine/non-manual occupations (reference), **Model 3:** individuals with lower versus higher education (reference, since 2004); all models adjusted for age, sex, and year of stroke.

After adjusting for age, sex, and stroke year, vascular risk profiles differed significantly by ethnicity (Fig. 2A, Model 1). Compared to White participants, diabetes was around twice as common in Black Caribbean (aPR: 2.23 [95% CI:2.04–2.43]) and Black African participants (1.92 [1.73–2.13]), while hypertension was 29% and 47% and overweight/obesity 13% and 23% more prevalent respectively. Conversely, myocardial infarction, smoking, and AF were markedly less common, with AF prevalence 43% lower in Black Caribbean (aPR 0.57 [0.48–0.68]) and 34% lower in Black African participants (0.66 [0.51–0.80]).

Diabetes prevalence was 23% higher in participants with routine/manual occupations (vs non-routine/non-manual; aPR 1.23 [1.13–1.34]) and 21% higher in those with lower education (vs higher education; aPR 1.21 [1.09–1.37]; Fig. 2A, Model 2 & 3). Hypertension, overweight/obesity, and smoking were also significantly more prevalent in lower-SES participants.

There was only marginal attenuation in risk factor estimates in models mutually adjusted for ethnicity and either occupation or education (Model 4 & 5, Table S7/Supplement); Further models including interactions between ethnicity and occupation or education were not significant, apart from an interaction between Black African ethnicity and manual/routine occupation being associated with less hypertension (Table S8/Supplement).

Reflecting these risk factor profiles, Black Caribbean, Black African, and lower-SES participants had more strokes due to small vessel occlusion (SVO) than their counterparts (Table S9 A & B/Supplement). Black Caribbean and Black African participants had fewer cardioembolic strokes, but primary intracerebral haemorrhages were most common among Black African participants.

### Trends in risk factor prevalence over time

**Fig. 3:**
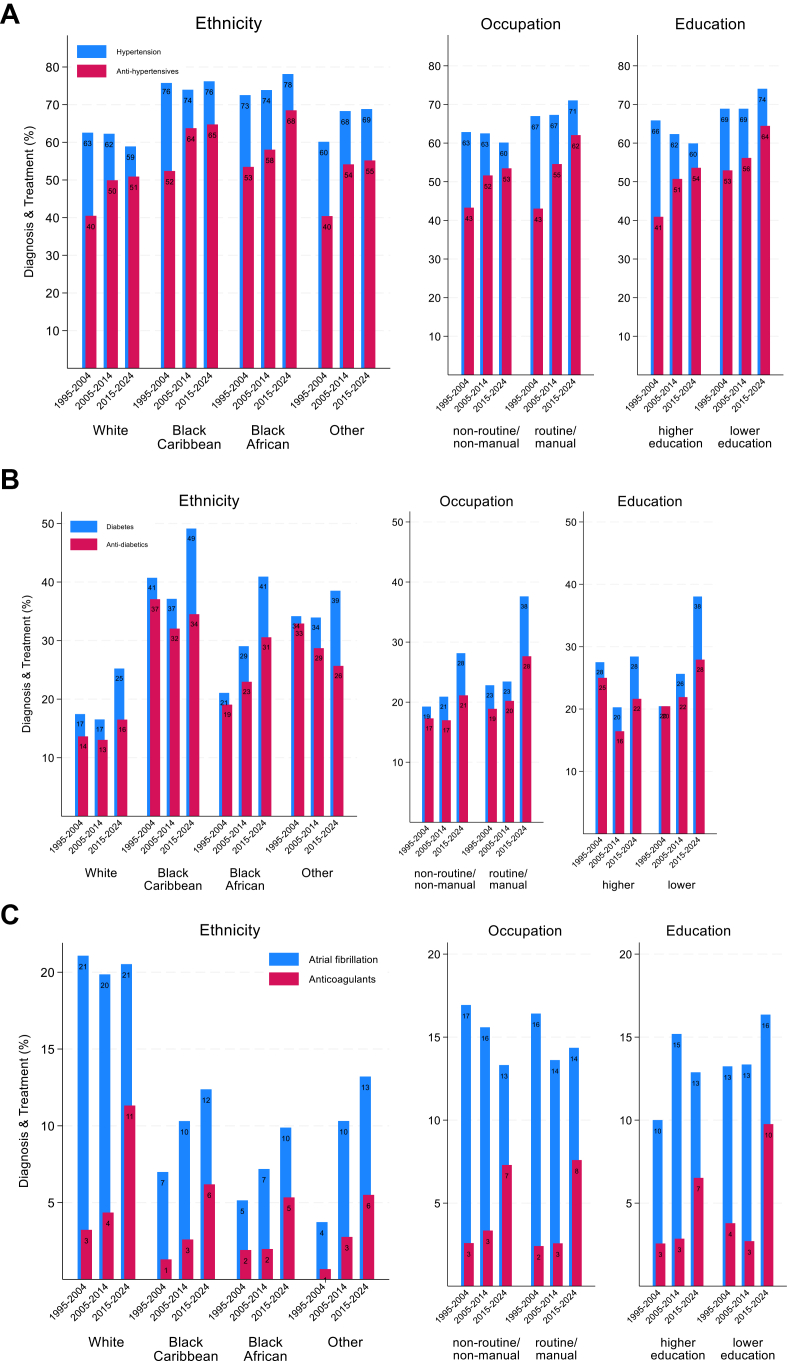
**Trends in pre-stroke vascular risk factor diagnoses and treatment, stratified by ethnicity, occupation, and education. Panel A: Hypertension & Anti-hypertensives∗. p-values for trends across cohorts** calculated using Cochran–Armitage test of trend: **Hypertension:** White 0.041, Black Caribbean 0.954, Black African 0.074, Other 0.082; non-routine/non-manual 0.302; routine/manual 0.021; higher education 0.183, lower education 0.025; **Anti-hypertensives** (including only those with hypertension): White <0.001, Black Caribbean <0.001, Black African <0.001, Other 0.002; non-routine/non-manual <0.001, routine/manual <0.001; higher education <0.001, lower education 0.005. **Panel B: Diabetes & Anti-diabetics∗. p-value for trends across cohorts** calculated using Cochran–Armitage test of trend: **Diabetes:** White <0.001, Black Caribbean 0.006, Black African <0.001, Other 0.275; non-routine/non-manual <0.001, routine/manual <0.001; higher education <0.001, lower education <0.001; **Anti-diabetics** (including only those with diabetes): White <0.001, Black Caribbean <0.001, Black African 0.048, Other <0.001; nonroutine/non-manual <0.001, routine/manual 0.001; higher education 0.031, lower education, 0.001. **Panel C: Atrial fibrillation & Anticoagulants∗. p-values for trends across cohorts** calculated using Cochran–Armitage test of trend: **Atrial fibrillation:** White 0.633, Black Caribbean 0.014, Black African 0.030, Other 0.002; non-routine/non-manual 0.042, routine/manual 0.068; higher education 0.220, lower education 0.091; **Anticoagulants** (including only those with atrial fibrillation): White <0.001, Black Caribbean 0.005, Black African 0.067, Other 0.138; non-routine/non-manual <0.001, routine/manual <0.001; higher education <0.001, lower education <0.00; **∗**Percentages of treatment rates in figure refer to the overall study population, rather than only those with the respective risk factor diagnosis as in the manuscript text, in order to show them side by side in the same diagram with risk factor diagnosis.

Over time, hypertension declined in White participants but remained stable in Black Caribbean and increased (non-significantly) in Black African participants (Fig. 3). Diabetes increased in all ethnic groups, but this increase was most pronounced in Black African participants (21% in 1995–2004 to 41% in 2015–2024), with the highest prevalence observed in Black Caribbean individuals (49% in 2015–2024). AF diagnoses increased in ethnic minority but not White participants (Fig. 3).

In adjusted analyses, associations between Black African ethnicity and hypertension or diabetes became stronger over time (hypertension: aPR 1.37 [1.2–1.52] in 1995–2004 versus 1.54 [1.45–1.64] in 2015–2024; diabetes: 1.50 [1.09–2.07] versus 1.88 [1.65–2.15], Table S11/Supplement); the strength of the age-adjusted association between lower AF prevalence and Black ethnicity declined but remained significant for Black Caribbean participants (aPR 0.54 [0.29–0.73] in 2015–2024).

Diabetes diagnoses increased in all socioeconomic groups but more strongly in those with routine/manual occupation (23%–38%) or lower education (20%–38%, Fig. 3). Hypertension diagnoses increased in lower-SES participants but not their counterparts (Fig. 3) and associations between hypertension and lower SES became stronger over time (Table S10/Supplement).

Interaction terms between ethnicity and cohort or occupation and cohort were not statistically significant (Table S11/Supplement).

### Inequalities in primary prevention treatments and trends over time

The proportion of stroke patients with at least one diagnosed, but untreated pre-stroke vascular risk factor decreased marginally from 38% in 1995–2004 to 36% in 2015–2024 (Table 1). This proportion was higher in White participants (39%) than Black Caribbean (37%) or Black African participants (35%, Table 2); over time it decreased in White participants from 40% to 35% (p < 0.001), increased in Black Caribbean (33%–40%, p = 0.046) and remained stable in Black African participants (33%–34%, p = 0.939, Table S5A/Supplement). The proportion with at least one diagnosed untreated vascular risk factor was higher in participants with routine/manual occupations than their counterparts (Table 3) but decreased over time in this subgroup (Table S5B/Supplement).

Between 1995–2004 and 2015–2024, treatment rates increased for hypertension (63%–83%), hypercholesterolaemia (68%–80%), and AF (anticoagulation: 15%–51%), while antidiabetic treatment decreased (84%–69%, Table 1). Similar trends were observed in all subgroups (Fig. 3). In participants with AF, the rate of anticoagulation declined with age, while the rate of antiplatelet treatment increased (Table S12/Supplement).

In adjusted analyses, Black Caribbean and Black African participants had higher treatment rates for hypertension and diabetes than White participants (Fig. 2B), while antithrombotic treatment (in those with previous TIA or MI) was lower in Black African participants (aPR 0.82 [0.70–0.95]). Stratified by cohorts, the strength of the association between Black Caribbean ethnicity and antihypertensive or anti-diabetic treatment declined, while it increased for Black African ethnicity (Table S10/Supplement). All other associations between treatment rates and ethnicity or SES were non-significant. Including ethnicity and education or occupation in mutually adjusted models (Model 4 & 5), had only marginal impact on the reported associations (Table S7/Supplement).

## Discussion

In this large population-based study of people with stroke spanning three decades, vascular risk factor profiles differed significantly by ethnicity and socioeconomic status; the underlying risk factor burden, particularly hypertension and diabetes, was higher and rising faster in Black Caribbean, Black African, and lower socioeconomic groups, contributing to widening inequalities in stroke incidence. Over a third of strokes continued to occur in people with diagnosed but untreated risk factors and a rising proportion had no pre-stroke risk factor diagnosis, calling for universal as well as targeted improvements in risk factor detection and management.

While ethnic and socioeconomic inequalities in risk factor profiles have been investigated,^3^^,^^4^ this study adds to the existing evidence in several ways. First, with 30 years of continuous data collection, this study highlights widening inequalities between ethnic and socioeconomic groups, particularly for hypertension and diabetes, thereby identifying targets for primary prevention programmes to reduce health inequalities. Second, while previous studies focused on either ethnic or socioeconomic inequalities, this study includes both and shows that these characteristics, although often similarly associated with risk factors, act independently rather than one driving the other. Third, despite the UK's universal healthcare provision, pre-stroke risk factor detection and treatment rates in this population-based study remain suboptimal.

In many countries, age-adjusted stroke incidence declined in recent decades due to advances in primary prevention but contrasts with reported increases in vascular risk factor burden.^18^, ^19^, ^20^ Our study aligns with those reports, showing increasing proportions of participants with three or more risk factors. As well as true increases in prevalence, some increase will be due to improved detection, such as for the observed steep rise in hypercholesterolaemia. Combined with generally improved treatment rates as reported here and incentivised by UK's pay-for-performance policies,^2^ these might explain declining stroke incidence despite rising risk factor burden. Additionally, improvements in lifestyle factors, e.g. reduced smoking rates, and factors not included here, e.g. improving physical activity levels among UK adults,^18^ might play a role.

However, these overall figures mask large inequalities: we observed higher rates of hypertension and diabetes, prime drivers of stroke incidence inequalities,^4^^,^^21^^,^^22^ in ethnic minority and lower-SES participants and reflected in their higher rates of SVO-related strokes.^20^ These inequalities widened over time; and while widening risk factor inequalities might be influenced by changes in age profiles to some extent, they risk further increases in stroke incidence inequalities. Together with higher proportions of strokes without pre-stroke risk factor diagnoses in Black African participants, this demonstrates a need for awareness and screening campaigns targeting high-risk populations. Reducing health inequalities through targeted approaches has been prioritised in both the UK government's 10-year Health Plan for England^23^ and the national primary care audit CVDPREVENT.^24^ However, the UK's NHS Health Checks programme, offering vascular assessments for adults aged 40–74, reportedly has low uptake particularly in more deprived groups.^25^ Furthermore, the younger-age onset of hypertension and diabetes^4^ and subsequently first stroke in Black ethnic groups calls for earlier checks in certain populations; the current start of vascular health checks in the UK at age 40 might be too late and miss primary prevention opportunities in high-risk groups. Higher prevalence of overweight/obesity in ethnic minority and lower-SES participants additionally underlines the importance of lifestyle and behavioural approaches. Addressing inequalities in vascular health and achieving more equitable benefits of public health programmes requires further identification of groups most at risk and understanding their barriers to uptake. In London, initiatives such as *Vital 5* were developed through integrated clinical and community strategies with a focus on reducing health inequalities in deprived or ethnic minority communities, e.g. through improved blood pressure control and lifestyle changes.^26^

We observed mostly equitable treatment rates between ethnic and socioeconomic groups in line with several^6^^,^^27^ but not all^28^ previous studies. The UK's universal healthcare provision might play a role, lowering direct financial treatment costs; and the observed higher treatment rates for hypertension and diabetes in Black African and Caribbean participants, as well as their changes over time, could reflect severity levels of those risk factors in the respective groups. However, while we had no data on risk factor severity or control rates, inequalities in risk factor control have been reported, including specifically for this study's catchment area in London.^6^^,^^27^^,^^29^^,^^30^ The discrepancy between equitable or higher treatment but diverging control rates calls for patient-centred strategies to improve medication adherence and targeted, more intensive monitoring, mindful of any potential structural racism. Interventions, based on trust-building, culturally-tailored communications, and shared-decision making, have been shown to be effective.^4^ For example, a one-year project in London, delivered through primary care, successfully eradicated a 12%-gap in hypertension control between ethnic minority and White patients.^31^ In a US randomised controlled trial, health promotion through barbershops linked to medication management led to large and sustained blood pressure reductions in Black men.^32^

In this population-based cohort study, both ethnicity and SES were independently associated with vascular risk factors, and SES did not explain ethnic inequalities. Previous evidence on the role of SES in explaining ethnic inequalities in stroke risk factors are scarce and conflicting.^10^ The UK's universal healthcare provision might reduce the impact of SES on health and healthcare inequalities, as our finding of equitable treatment rates indicate. However, there were significant and worsening socioeconomic inequalities in risk factor prevalence, suggesting that factors other than direct financial barriers to healthcare access are important, including upstream social determinants of health, e.g. early childhood factors, education, psychosocial stress, housing quality, or environmental exposure.^10^ Additionally, in this study, the association between ethnic minority and socioeconomic status was nuanced: Black African participants had higher and Black Caribbean participants lower proportions of high education and non-routine/non-manual occupations than White participants. This suggests that apart from potential non-modifiable biological factors, social and cultural rather than socioeconomic factors might play an important role in driving ethnic risk factor inequalities and need to be identified in future research. Addressing these inequalities will require multifaceted interventions and integrated care approaches: person-centred care, provided by joined up-services, will need to address physical but also mental health and social needs, with a focus on prevention and supporting people in the community.^33^

As in previous studies,^8^ Black minority participants had lower rates of AF. This was unlikely due to selective under-diagnosis, as rates of newly diagnosed AF at stroke were similar across groups. Over time, AF became more prevalent in Black participants, calling for AF awareness campaigns among Black people but also healthcare providers, especially given AF's silent and often paroxysmal nature.

AF management changed significantly during this study: a 2007 meta-analysis^34^ found anticoagulation superior to aspirin in preventing stroke; DOACs widely replaced Vitamin K antagonists, requiring less monitoring; and the CHA_2_DS_2_-VASc-score superseded the CHADS_2_-score, widening eligibility for anticoagulation. However, despite significant increases, anticoagulation remained widely underused across all groups, especially in the older age groups. While we had no data on contraindications, these are unlikely to justify 48% non-anticoagulated, high-risk people who then proceeded to having stroke. Some previous studies reported lower anticoagulation rates in ethnic minority and lower-SES participants, as well as higher usage of Warfarin compared to DOAC.^35^^,^^36^ We found no significant inequalities in anticoagulation rates but could not investigate the relative usage of Warfarin or DOAC due to a lack of data.

This study has several strengths. It benefits from a continuous and prospective collection of sociodemographic and clinical data over 30-years in a population-based cohort. This facilitates analyses of long-term trends of inequalities in vascular risk factors and treatments, reflecting prevalence trends, diagnostic efforts, and implementation of evidence-based guidelines. Detailed ethnic and socioeconomic data for this large, multi-ethnic cohort enabled us to investigate the effect of SES on ethnic inequalities and stratification into different Black ethnic groups, often combined in studies and masking distinct cultural identities and migration histories. Additionally, the UK's universal healthcare provision provides a different lens through which to study health and healthcare inequalities compared to studies set in insurance-based systems.

However, the following limitations should also be considered. This study lacked data on the severity of risk factors, medication adherence, or control rates, which might differ systematically between ethnic and socioeconomic groups and between stroke patients and the general population. Vascular risk factor data included only diagnosed conditions, rather than true prevalence. Additionally, risk factor prevalence in this cohort cannot be generalised to a stroke-free population, despite general trends being broadly similar^18^; they do however demonstrate important drivers of stroke risk.

Rates of missingness for vascular risk factors and treatments were low but higher for SES indicators. However, sensitivity analyses using multiple imputation methods did not show meaningfully different results and confirmed the robustness of the presented findings.

Finally, while the population-based design of this study reduces selection bias and increases representativeness, the results will be influenced by the study setting's specific demography. However, our stratified and adjusted analyses allow interpretations for populations with other demographic makeups.

In conclusion, inequalities in vascular risk factor profiles are significant and widening; hypertension and diabetes, major drivers of stroke incidence inequalities, increased disproportionately in ethnic minority and lower-SES participants, which urgently requires targeted and culturally tailored primary prevention. The current start of vascular health checks in the UK at age 40 might miss primary prevention opportunities in high-risk groups who often experience first stroke at younger ages. One in ten strokes occur without prior risk factor diagnosis, and over a third in people with at least one diagnosed but untreated risk factor, highlighting a need for universal as well as targeted improvements in risk factor detection and management.

## Contributors

ESE, IJM, and MDLOC conceptualised and designed the study. ESE undertook the formal analysis and visualisation and wrote the original draft. All other authors reviewed and edited subsequent versions. ESE and CPR have accessed and verified the data. EL additionally contributed to data curation, MDLOC and IJM to supervision, MDLOC to validation, and AB, CDAW, MDLOC and IJM to funding acquisition. All authors had full access to all the data in the study and had final responsibility for the decision to submit for publication. All authors read and approved the final version.

We thank the stroke survivors, their families and carers, the staff in the stroke services facilitating the study, and all fieldworkers who have collected data over the course of the study.

## Data sharing statement

Requests for data access for academic use should be made to the SLSR team, where data will be made available subject to academic review and acceptance of a data-sharing agreement.

## Declaration of interests

Nothing to declare.
