# Supplementary material for: Ethnic and socioeconomic inequalities in stroke risk factors and primary prevention: the South London Stroke Register cohort study 1995–2024
Source: eClinicalMedicine. 2026 Jul 9;97:104061. doi: 10.1016/j.eclinm.2026.104061 (PMC13380083; doi:10.1016/j.eclinm.2026.104061)
Supplement: Supplement Tables S1–S12 [file mmc1.docx]

**Supplementary Material**

[**Table S1: Number of missing values (% of total study population (N=8,515)) for each variable** 2](#_Toc232754096)

[**Table S2: Baseline characteristics of the study population (N=8,515) with and without missing occupation or education** 3](#_Toc232754097)

[**Table S3: Adjusted prevalence ratios of pre-stroke risk factor diagnoses & treatments between ethnic and socioeconomic groups using imputed datasets** 4](#_Toc232754098)

[**Table S4: Characteristics of the study population and SLSR participants excluded due to missing socio-demographic indicators (ethnicity, occupation, and education)** 5](#_Toc232754099)

[**Table S5A: Trends in characteristics of the study population, stratified by ethnicity** 6](#_Toc232754100)

[**Table S5B: Trends in characteristics of the study population, stratified by occupation and education** 7](#_Toc232754101)

[**Table S6: Prevalence ratios of routine/manual occupations and lower education among ethnic minorities vs white individuals** 8](#_Toc232754102)

[**Table S7: Adjusted prevalence ratio (95%CI) for pre-stroke VRFs and appropriate primary prevention treatment, mutually adjusted for ethnicity and occupation (model 4) or ethnicity and education (model 5)** 9](#_Toc232754103)

[**Table S8: Adjusted prevalence ratio (95%CI) for pre-stroke VRFs and appropriate primary prevention treatment, including interaction terms between ethnicity and occupation (model 6) or ethnicity and education (model 7)** 10](#_Toc232754104)

[**Table S9A: Risk factor profile, primary prevention treatment and stroke type, stratified by ethnicity** 13](#_Toc232754105)

[**Table S9B: Risk factor profile, primary prevention treatment and stroke type, stratified by occupation and education** 14](#_Toc232754106)

[**Table S10: Adjusted prevalence rate ratio (95%CI) of pre-stroke VRFs and primary prevention treatments among ethnic minority groups (model 1) and participants with routine/manual occupation (model 2) and lower educational attainment (model 3), stratified by cohort** 15](#_Toc232754107)

[**Table S11: Adjusted prevalence ratio (95%CI) for pre-stroke VRFs with interaction terms between ethnicity and cohort (Model 8) or occupation and cohort (Model 9)** 17](#_Toc232754108)

[**Table S12: Characteristics of the study population (N=8,515), 1995-2024, stratified by sex and age groups** 19](#_Toc232754109)

# **Table S1: Number of missing values (% of total study population (N=8,515)) for each variable**

| Variable | N (%) of missing values | collected since |
| --- | --- | --- |
| Age | 0 (0.0%) | 1995 |
| Sex | 0 (0.0%) | 1995 |
| Year of stroke | 0 (0.0%) | 1995 |
| Ethnicity | 95 (1.1%) | 1995 |
| Occupation | 1,982 (23.3%) | 1995 |
| Education | 1,607 (27.3%) | 2004 |
| Hypertension | 348 (4.1%) | 1995 |
| Antihypertensive treatment | 335 (4.0%) | 1995 |
| Diabetes | 272 (3.2%) | 1995 |
| Diabetes treatment | 222 (2.6%) | 1995 |
| Atrial fibrillation | 404 (4.7%) | 1995 |
| Newly diagnosed atrial fibrillation | 1,499 (23.6%) | 2002 |
| Anticoagulants | 318 (3.8%) | 1995 |
| Antiplatelets | 318 (3.8%) | 1995 |
| Hypercholesterolaemia | 593 (7.9%) | 1998 |
| Cholesterol-lowering treatment | 716 (8.5%) | 1995 |
| Myocardial infarction | 401 (4.7%) | 1995 |
| Transient ischaemic attack | 388 (4.6%) | 1995 |
| Smoking | 836 (9.9%) | 1995 |
| Body Mass Index | 2,250 (34.0%) | 2001 |
| Ischaemic or haemorrhagic stroke | 177 (2.1%) | 1995 |
| TOAST classification | 978 (13.7%) | 1999 |

# **Table S2: Baseline characteristics of the study population (N=8,515) with and without missing occupation or education**

|  | Occupation | | | Education (from 2004) | | |
| --- | --- | --- | --- | --- | --- | --- |
|  | **Not missing** | **missing** | **p-value** | **Not missing** | **missing** | **p-value** |
|  | **N=6,533** | **N=1,982** |  | **N=4,281** | **N=1,607** |  |
| Cohort |  |  | **<0.001** |  |  | **<0.001** |
| 1995-2004 | 2,400 (36.7%) | 507 (25.6%) |  | 140 (3.3%) | 140 (8.7%) |  |
| 2005-2014 | 2,027 (31.0%) | 670 (33.8%) |  | 2,013 (47.0%) | 684 (42.6%) |  |
| 2015-2024 | 2,106 (32.2%) | 805 (40.6%) |  | 2,128 (49.7%) | 783 (48.7%) |  |
| Age, years (median, IQR) | 69.5 (58.3-79.5) | 74.6 (62.0-83.5) | **<0.001** | 67.3 (56.0-78.4) | 73.6 (62.8-82.3) | **<0.001** |
| Sex, female | 2,904 (44.5%) | 1,148 (57.9%) | **<0.001** | 1,880 (43.9%) | 847 (52.7%) | **<0.001** |
| Ethnicity |  |  | **<0.001** |  |  | **<0.001** |
| White | 4,024 (62.5%) | 1,231 (62.2%) |  | 2,339 (55.1%) | 950 (59.6%) |  |
| Black Caribbean | 1,002 (15.6%) | 301 (15.2%) |  | 725 (17.1%) | 267 (16.8%) |  |
| Black African | 902 (14.0%) | 220 (11.1%) |  | 768 (18.1%) | 195 (12.2%) |  |
| Other | 514 (8.0%) | 226 (11.4%) |  | 413 (9.7%) | 182 (11.4%) |  |
| Occupation |  |  |  |  |  | **0.003** |
| Non-routine/non-manual | 2,569 (39.3%) |  |  | 1,686 (44.2%) | 205 (37.4%) |  |
| Routine/manual | 3,964 (60.7%) |  |  | 2,132 (55.8%) | 343 (62.6%) |  |
| Education^1^ |  |  | **<0.001** |  |  |  |
| higher education | 2,176 (56.5%) | 218 (46.5%) |  | 2,379 (55.6%) |  |  |
| lower education | 1,678 (43.5%) | 251 (53.5%) |  | 1,902 (44.4%) |  |  |
| Number of VRFs |  |  | **0.22** |  |  | **<0.001** |
| no VRF | 482 (7.6%) | 117 (6.5%) |  | 399 (9.4%) | 84 (5.7%) |  |
| 1 or 2 VRFs | 3,628 (57.0%) | 1,056 (58.6%) |  | 2,204 (52.2%) | 830 (56.4%) |  |
| more than 2 VRFs | 2,254 (35.4%) | 628 (34.9%) |  | 1,623 (38.4%) | 557 (37.9%) |  |
| ≥1 untreated VRF | 2,017 (34.3%) | 694 (41.2%) | **<0.001** | 1,222 (31.9%) | 563 (40.6%) | **<0.001** |
| Hypertension | 4,159 (65.5%) | 1,231 (67.6%) | **0.11** | 2,779 (65.5%) | 1,047 (70.0%) | **0.001** |
| antihypertensive treatment* | 3,008 (74.1%) | 898 (74.6%) | **0.71** | 2,195 (79.8%) | 801 (77.6%) | **0.15** |
| Diabetes mellitus | 1,638 (25.7%) | 549 (29.3%) | **0.002** | 1,170 (27.6%) | 489 (31.5%) | **0.003** |
| diabetes treatment* | 1,280 (79.6%) | 357 (66.2%) | **<0.001** | 900 (77.9%) | 322 (66.4%) | **<0.001** |
| Atrial fibrillation | 952 (15.1%) | 380 (21.1%) | **<0.001** | 598 (14.3%) | 299 (20.3%) | **<0.001** |
| Newly diagnosed atrial fibrillation | 224 (5.9%) | 78 (7.1%) | **0.15** | 180 (5.2%) | 90 (8.0%) | **<0.001** |
| Anticoagulants if AF* | 254 (27.4%) | 131 (34.9%) | **0.007** | 215 (36.3%) | 112 (37.8%) | **0.64** |
| Anticoagulants if high-risk AF* | 206 (32.2%) | 112 (39.2%) | **0.04** | 196 (38.1%) | 100 (37.6%) | **0.9** |
| antiplatelets if AF* | 374 (40.4%) | 131 (34.9%) | **0.067** | 233 (39.3%) | 115 (38.9%) | **0.9** |
| Hypercholesterolaemia | 1,684 (31.4%) | 535 (32.7%) | **0.33** | 1,534 (36.4%) | 520 (35.2%) | **0.4** |
| cholesterol-lowering treatment* | 1,254 (76.2%) | 405 (77.0%) | **0.72** | 1,182 (77.9%) | 387 (75.7%) | **0.32** |
| Myocardial infarction | 678 (10.7%) | 233 (12.9%) | **0.009** | 425 (10.1%) | 192 (13.1%) | **0.002** |
| TIA | 687 (11.0%) | 169 (9.5%) | **0.066** | 390 (9.3%) | 140 (9.6%) | **0.77** |
| antithrombotics in TIA or MI* | 781 (65.1%) | 235 (66.2%) | **0.7** | 527 (72.5%) | 199 (67.0%) | **0.079** |
| Smoking, current or ex | 3,672 (58.7%) | 697 (49.8%) | **<0.001** | 2,288 (54.9%) | 560 (53.0%) | **0.27** |
| pre-stroke BMI≥25^2^ | 2,114 (57.9%) | 387 (49.0%) | **<0.001** | 1,900 (58.9%) | 387 (49.2%) | **<0.001** |
| Stroke type |  |  | **<0.001** |  |  | **<0.001** |
| Haemorrhagic stroke | 1,101 (17.1%) | 485 (25.3%) |  | 658 (15.4%) | 418 (26.2%) |  |
| Ischaemic stroke | 5,321 (82.9%) | 1,431 (74.7%) |  | 3,617 (84.6%) | 1,177 (73.8%) |  |
| TOAST^3^ |  |  | **<0.001** |  |  | **<0.001** |
| LAA | 455 (9.7%) | 107 (7.0%) |  | 398 (10.5%) | 87 (6.1%) |  |
| CE | 957 (20.3%) | 349 (23.0%) |  | 708 (18.8%) | 359 (25.2%) |  |
| SVO | 1,104 (23.5%) | 198 (13.0%) |  | 881 (23.3%) | 176 (12.4%) |  |
| OTH/UND | 1,354 (28.8%) | 457 (30.1%) |  | 1,172 (31.0%) | 410 (28.8%) |  |
| PICH | 609 (12.9%) | 317 (20.9%) |  | 466 (12.3%) | 302 (21.2%) |  |
| SAH | 224 (4.8%) | 90 (5.9%) |  | 151 (4.0%) | 90 (6.3%) |  |

Summary statistics are count (%); Percentages refer to those with known value as denominator; when indicated (*) referring to those with relevant VRF diagnosis; p-value for trend across cohorts was calculated using Cochran-Armitage test of trend for categorical variables.

**Abbreviations:** VRFs: Vascular risk factors, BMI: body mass index, TOAST classification: Trial of Org 10172 in Acute Stroke Treatment classification, LAA: large artery atherosclerosis, CE: cardioembolic, SVO: small vessel occlusion, Oth/UND: other or undefined ischaemic stroke, PICH: primary intracerebral haemorrhage, SAH: subarachnoid haemorrhage ^1^education recorded since 2004, ^2^ “BMI” recorded since 2001, ^3^ “TOAST classification” collected since 1999

# **Table S3: Adjusted prevalence ratios of pre-stroke risk factor diagnoses & treatments between ethnic and socioeconomic groups using imputed datasets**

|  | Ethnicity (vs White) | | Occupation (vs non-manual) /education (vs higher) | |
| --- | --- | --- | --- | --- |
| Hypertension | Black Caribbean | 1.29 (1.20-1.39) | routine/manual | 1.09 (1.03-1.16) |
|  | Black African | 1.46 (1.35-1.59) | lower education | 1.06 (0.99-1.14) |
|  | Other | 1.20 (1.09-1.33) |  |  |
| Diabetes | Black Caribbean | 2.22 (2.00-2.47) | routine/manual | 1.22 (1.10-1.35) |
|  | Black African | 1.91 (1.68-2.17) | lower education | 1.21 (1.08-1.36) |
|  | Other | 1.94 (1.69-2.23) |  |  |
| Atrial fibrillation | Black Caribbean | 0.57 (0.48-0.69) | routine/manual | 0.94 (0.83-1.07) |
|  | Black African | 0.67 (0.53-0.83) | lower education | 0.78 (0.67-0.92) |
|  | Other | 0.65 (0.51-0.82) |  |  |
| Hypercholesterolaemia | Black Caribbean | 1.15 (1.02-1.28) | routine/manual | 1.14 (1.04-1.26) |
|  | Black African | 1.09 (0.96-1.23) | lower education | 0.99 (0.89-1.09) |
|  | Other | 1.18 (1.03-1.36) |  |  |
| Myocardial infarction | Black Caribbean | 0.80 (0.65-0.97) | routine/manual | 1.08 (0.92-1.27) |
|  | Black African | 0.69 (0.54-0.88) | lower education | 0.98 (0.81-1.19) |
|  | Other | 1.04 (0.83-1.31) |  |  |
| TIA | Black Caribbean | 0.95 (0.78-1.15) | routine/manual | 1.04 (0.88-1.21) |
|  | Black African | 0.78 (0.61-1.01) | lower education | 0.95 (0.77-1.17) |
|  | Other | 0.88 (0.68-1.15) |  |  |
| Smoking | Black Caribbean | 0.79 (0.74-0.85) | routine/manual | 1.18 (1.12-1.25) |
|  | Black African | 0.35 (0.31-0.39) | lower education | 1.20 (1.12-1.28) |
|  | Other | 0.63 (0.57-0.69) |  |  |
| BMI>=25 | Black Caribbean | 1.15 (1.05-1.26) | routine/manual | 1.16 (1.07-1.25) |
|  | Black African | 1.23 (1.12-1.35) | lower education | 1.13 (1.04-1.23) |
|  | Other | 0.86 (0.75-0.99) |  |  |
| Antihypertensives | Black Caribbean | 1.13 (1.04-1.22) | routine/manual | 1.01 (0.95-1.07) |
|  | Black African | 1.19 (1.09-1.30) | lower education | 0.99 (0.92-1.07) |
|  | Other | 1.06 (0.96-1.18) |  |  |
| Anti-diabetes med | Black Caribbean | 1.15 (1.06-1.26) | routine/manual | 1.01 (0.94-1.10) |
|  | Black African | 1.17 (1.05-1.30) | lower education | 1.03 (0.93-1.14) |
|  | Other | 1.13 (1.01-1.27) |  |  |
| Cholesterol-lowering med | Black Caribbean | 1.00 (0.90-1.11) | routine/manual | 1.08 (0.99-1.18) |
|  | Black African | 0.96 (0.86-1.09) | lower education | 1.08 (0.99-1.19) |
|  | Other | 0.99 (0.87-1.13) |  |  |
| Anticoagulants if AF | Black Caribbean | 0.90 (0.70-1.15) | routine/manual | 0.96 (0.81-1.14) |
|  | Black African | 0.91 (0.70-1.17) | lower education | 1.11 (0.91-1.36) |
|  | Other | 0.81 (0.59-1.11) |  |  |
| Antithrombotics if MI or TIA | Black Caribbean | 0.98 (0.83-1.15) | routine/manual | 0.91 (0.81-1.03) |
|  | Black African | 0.79 (0.66-0.95) | lower education | 1.04 (0.87-1.24) |
|  | Other | 0.88 (0.72-1.08) |  |  |

All Poisson regression models additionally adjusted for age, sex, and year of stroke; Abbreviations: AF: atrial fibrillation, MI: Myocardial infarction, TIA: transient ischaemic attack, BMI: body mass index

# **Table S4: Characteristics of the study population and SLSR participants excluded due to missing socio-demographic indicators (ethnicity, occupation, and education)**

|  | SLSR participants included in the study | SLSR participants excluded | p-value |
| --- | --- | --- | --- |
|  | N=8,515 | N=128 |  |
| Age, years (median, IQR) | 70.5 (58.9-80.5) | 69.3 (53.6-81.1) | 0.275 |
| Sex, female | 4,052 (47.6%) | 59 (46.4%) | 0.81 |
| Vascular risk factors |  |  |  |
| Hypertension | 5,390 (66.0%) | 76 (59.6%) | 0.2 |
| Diabetes mellitus | 2,187 (26.5%) | 31 (24.5%) | 0.66 |
| Atrial fibrillation | 1,332 (16.4%) | 20 (15.9%) | 0.9 |
| Hypercholesterolaemia | 2,219 (31.7%) | 27 (20.9%) | 0.032 |
| Myocardial infarction | 911 (11.2%) | 11 (8.4%) | 0.42 |
| TIA | 856 (10.7%) | 6 (4.7%) | 0.074 |
| Smoking, current or ex | 4,369 (57.0%) | 92 (72.2%) | 0.025 |
| pre-stroke BMI ≥ 25 | 2,501 (56.3%) | 37 (28.6%) | <0.001 |

Summary statistics are count (%); Percentages refer to those with known value as denominator

# **Table S5A: Trends in characteristics of the study population, stratified by ethnicity**

|  | White | | | | Black Caribbean | | | | Black African | | | | | Other | | | |
| --- | --- | --- | --- | --- | --- | --- | --- | --- | --- | --- | --- | --- | --- | --- | --- | --- | --- |
|  | **1995-2004** | **2005-2014** | **2015-2024** | **P (trend)** | **1995-2004** | **2005-2014** | **2015-2024** | **P (trend)** | **1995-2004** | **2005-2014** | **2015-2024** | **P (trend)** | **1995-2004** | | **2005-2014** | **2015-2024** | **P (trend)** |
|  | **N=2,155** | **N=1,693** | **N=1,407** |  | **N=352** | **N=435** | **N=516** |  | **N=182** | **N=318** | **N=622** |  | **N=170** | | **N=230** | **N=340** |  |
| Age, years (median, IQR) | 75.2 (65.4-82.7) | 74.5 (61.7-83.0) | 71.3 (59.9-81.2) | **<0.001** | 67.9 (60.7-75.1) | 71.0 (56.8-77.9) | 69.6 (59.5-82.1) | **<0.001** | 56.6 (46.5-66.4) | 58.3 (49.1-68.6) | 59.8 (51.8-71.6) | **<0.001** | 65.7 (56.4-74.1)) | | 66.1 (54.2-77.5) | 64.5 (53.0-75.9) | **0.6656** |
| Sex, female | 1,118 (51.9%) | 828 (48.9%) | 610 (43.4%) | **<0.001** | 159 (45.2%) | 207 (47.6%) | 262 (50.8%) | **0.100** | 87 (47.8%) | 138 (43.4%) | 267 (42.9%) | **0.299** | 68 (40.0%) | | 108 (47.0%) | 156 (45.9%) | **0.277** |
| Occupation |  |  |  |  |  |  |  |  |  |  |  |  |  | |  |  |  |
| higher occupation | 577 (32.8%) | 540 (42.5%) | 517 (52.2%) | **<0.001** | 50 (16.2%) | 91 (27.3%) | 134 (37.1%) | **<0.001** | 70 (47.3%) | 103 (41.9%) | 224 (44.1%) | **0.692** | 42 (31.1%) | | 73 (47.1%) | 105 (46.9%) | **0.007** |
| lower occupation | 1,184 (67.2%) | 732 (57.5%) | 474 (47.8%) | **<0.001** | 258 (83.8%) | 242 (72.7%) | 227 (62.9%) | **<0.001** | 78 (52.7%) | 143 (58.1%) | 284 (55.9%) | **0.692** | 93 (68.9%) | | 82 (52.9%) | 119 (53.1%) | **0.007** |
| Education |  |  |  |  |  |  |  |  |  |  |  |  |  | |  |  |  |
| higher education | 22 (19.0%) | 613 (49.3%) | 602 (59.8%) | **<0.001** | 10 (33.3%) | 159 (46.9%) | 203 (55.6%) | **0.003** | 8 (44.4%) | 174 (69.0%) | 339 (67.5%) | **0.527** | 4 (23.5%) | | 101 (60.1%) | 136 (58.9%) | **0.166** |
| lower education | 94 (81.0%) | 630 (50.7%) | 404 (40.2%) | **<0.001** | 20 (66.7%) | 180 (53.1%) | 162 (44.4%) | **0.003** | 10 (55.6%) | 78 (31.0%) | 163 (32.5%) | **0.527** | 13 (76.5%) | | 67 (39.9%) | 95 (41.1%) | **0.166** |
| ≥1 untreated VRF | 785 (40.4%) | 514 (33.6%) | 432 (35.0%) | **<0.001** | 109 (33.4%) | 125 (31.6%) | 189 (39.6%) | **0.046** | 49 (32.9%) | 97 (36.1%) | 180 (33.7%) | **0.939** | 51 (35.2%) | | 63 (32.6%) | 117 (40.2%) | **0.200** |
| Hypercholesterol-aemia | 156 (14.1%) | 530 (32.2%) | 536 (39.7%) | **<0.001** | 29 (14.6%) | 141 (32.7%) | 231 (46.6%) | **<0.001** | 15 (11.8%) | 97 (31.9%) | 224 (37.4%) | **<0.001** | 24 (23.1%) | | 75 (33.5%) | 138 (42.6%) | **<0.001** |
| cholesterol-lowering treatment | 97 (68.8%) | 396 (75.1%) | 431 (80.4%) | **0.002** | 18 (69.2%) | 104 (73.8%) | 186 (80.5%) | **0.069** | 6 (50.0%) | 60 (61.9%) | 179 (79.9%) | **<0.001** | 17 (73.9%) | | 57 (76.0%) | 108 (78.3%) | **0.591** |
| Myocardial infarction | 264 (13.1%) | 167 (10.2%) | 190 (14.1%) | **0.591** | 22 (6.7%) | 28 (6.7%) | 68 (13.8%) | **<0.001** | 9 (5.2%) | 14 (4.6%) | 53 (8.8%) | **0.190** | 14 (8.7%) | | 19 (8.5%) | 49 (15.3%) | **0.015** |
| TIA | 287 (14.4%) | 158 (9.6%) | 147 (10.9%) | **<0.001** | 36 (10.8%) | 31 (7.2%) | 61 (12.3%) | **0.329** | 15 (8.6%) | 16 (5.2%) | 44 (7.4%) | **0.952** | 16 (9.9%) | | 18 (8.2%) | 27 (8.4%) | **0.617** |
| Antithrombotics in TIA/ MI | 277 (57.1%) | 223 (74.3%) | 213 (72.2%) | **<0.001** | 28 (54.9%) | 41 (73.2%) | 78 (70.9%) | **0.075** | 5 (28%) | 20 (67%) | 51 (59%) | **0.072** | 13 (54%) | | 23 (68%) | 44 (67%) | **0.349** |
| Smoking, current or ex | 1,335 (66.8%) | 1,038 (68.8%) | 777 (63.5%) | **0.115** | 186 (55.9%) | 216 (54.5%) | 218 (48.2%) | **0.028** | 45 (27.4%) | 94 (32.6%) | 128 (22.5%) | **0.029** | 82 (51.9%) | | 79 (38.3%) | 119 (41.6%) | **0.074** |
| pre-stroke BMI≥25 | 188 (46.9%) | 547 (54.6%) | 579 (52.8%) | **0.153** | 52 (57.8%) | 174 (64.7%) | 224 (58.2%) | **0.487** | 34 (72.3%) | 132 (66.7%) | 349 (70.2%) | **0.725** | 15 (40.5%) | | 65 (45.1%) | 126 (51.2%) | **0.130** |
| TOAST |  |  |  |  |  |  |  |  |  |  |  |  |  | |  |  |  |
| LAA | 78 (8.8%) | 155 (11.0%) | 132 (10.3%) | **0.329** | 9 (5.6%) | 50 (13.0%) | 27 (5.8%) | **0.224** | 5 (4.3%) | 23 (8.6%) | 39 (6.9%) | **0.688** | 8 (8.9%) | | 15 (7.7%) | 18 (5.7%) | **0.225** |
| CE | 233 (26.4%) | 352 (24.9%) | 313 (24.5%) | **0.331** | 27 (16.7%) | 51 (13.2%) | 78 (16.7%) | **0.622** | 13 (11.2%) | 38 (14.1%) | 82 (14.4%) | **0.431** | 8 (8.9%) | | 35 (17.9%) | 58 (18.2%) | **0.076** |
| SVO | 178 (20.2%) | 271 (19.2%) | 217 (17.0%) | **0.053** | 62 (38.3%) | 102 (26.4%) | 103 (22.1%) | **<0.001** | 33 (28.4%) | 64 (23.8%) | 133 (23.4%) | **0.327** | 22 (24.4%) | | 37 (18.9%) | 60 (18.9%) | **0.332** |
| OTH/UND | 220 (24.9%) | 424 (30.0%) | 373 (29.2%) | **0.052** | 35 (21.6%) | 111 (28.8%) | 163 (34.9%) | **0.001** | 25 (21.6%) | 84 (31.2%) | 180 (31.7%) | **0.072** | 20 (22.2%) | | 61 (31.1%) | 99 (31.1%) | **0.78** |
| PICH | 116 (13.1%) | 161 (11.4%) | 187 (14.6%) | **0.204** | 21 (13.0%) | 51 (13.2%) | 74 (15.8%) | **0.262** | 35 (30.2%) | 45 (16.7%) | 106 (18.7%) | **0.045** | 21 (23.3%) | | 39 (19.9%) | 62 (19.5%) | **0.483** |
| SAH | 58 (6.6%) | 52 (3.7%) | 57 (4.5%) | **0.045** | 8 (4.9%) | 21 (5.4%) | 22 (4.7%) | **0.794** | 5 (4.3%) | 15 (5.6%) | 28 (4.9%) | **0.961** | 11 (12.2%) | | 9 (4.6%) | 21 (6.6%) | **0.225** |

Summary statistics are count (%); Percentages refer to those with known value as denominator; when indicated (*) referring to those with relevant VRF diagnosis; p-value for trend across cohorts was calculated using Cochran-Armitage test of trend for categorical variables.

**Abbreviations:** VRFs: Vascular risk factors, BMI: body mass index, BI: Barthel Index, TOAST classification: Trial of Org 10172 in Acute Stroke Treatment classification, LAA: large artery atherosclerosis, CE: cardioembolic, SVO: small vessel occlusion, Oth/UND: other or undefined ischaemic stroke, PICH: primary intracerebral haemorrhage, SAH: subarachnoid haemorrhage ^1^education recorded since 2004, ^2^ “BMI” recorded since 2001, ^3^ “TOAST classification” collected since 1999

# **Table S5B: Trends in characteristics of the study population, stratified by occupation and education**

|  | Non-routine/non-manual occupation | | | | Routine/manual occupation | | | | Higher education | | | | Lower education | | | |
| --- | --- | --- | --- | --- | --- | --- | --- | --- | --- | --- | --- | --- | --- | --- | --- | --- |
|  | **1995-2004** | **2005-2014** | **2015-2024** | **P (trend)** | **1995-2004** | **2005-2014** | **2015-2024** | **P (trend)** | **1995-2004** | **2005-2014** | **2015-2024** | **P (trend)** | **1995-2004** | **2005-2014** | **2015-2024** | **P (trend)** |
|  | **N=757** | **N=819** | **N=993** |  | **N=1,643** | **N=1,208** | **N=1,113** |  | N=44 | N=1,054 | N=1,296 |  | N=138 | N=959 | N=832 |  |
| Age, years (median, IQR) | 72.0 (60.0-81.3) | 69.7 (56.5-80.6) | 65.6 (54.7-76.7) | **<0.001** | 72.5 (63.3-80.5) | 69.1 (57.6-79.4) | 66.1 (56.7-77.2) | **<0.001** | 65.0 (52.9-74.7) | 65.0 (53.1-77.1) | 62.4 (52.5-73.4) | **0.001** | 71.9 (62.1-79.3) | 73.0 (61.7-81.4) | 71.3 (59.8-81.2) | **0.688** |
| Sex, female | 413 (54.6%) | 389 (47.5%) | 446 (44.9%) | **<0.001** | 749 (45.6%) | 495 (41.0%) | 412 (37.0%) | **<0.001** | 17 (38.6%) | 444 (42.1%) | 579 (44.7%) | **0.155** | 58 (42.0%) | 472 (49.2%) | 325 (39.1%) | **0.002** |
| Ethnicity |  |  |  |  |  |  |  |  |  |  |  |  |  |  |  |  |
| White | 577 (78.1%) | 540 (66.9%) | 517 (52.8%) | **<0.001** | 1,184 (73.4%) | 732 (61.1%) | 474 (42.9%) | **<0.001** | 22 (50.0%) | 613 (58.5%) | 602 (47.0%) | **<0.001** | 94 (68.6%) | 630 (66.0%) | 404 (49.0%) | **<0.001** |
| Black Caribbean | 50 (6.8%) | 91 (11.3%) | 134 (13.7%) | **<0.001** | 258 (16.0%) | 242 (20.2%) | 227 (20.6%) | **<0.001** | 10 (22.7%) | 159 (15.2%) | 203 (15.9%) | **0.922** | 20 (14.6%) | 180 (18.8%) | 162 (19.7%) | **0.244** |
| Black African | 70 (9.5%) | 103 (12.8%) | 224 (22.9%) | **<0.001** | 78 (4.8%) | 143 (11.9%) | 284 (25.7%) | **<0.001** | 8 (18.2%) | 174 (16.6%) | 339 (26.5%) | **<0.001** | 10 (7.3%) | 78 (8.2%) | 163 (19.8%) | **<0.001** |
| Other | 42 (5.7%) | 73 (9.0%) | 105 (10.7%) | **0.064** | 93 (5.8%) | 82 (6.8%) | 119 (10.8%) | **0.735** | 4 (9.1%) | 101 (9.6%) | 136 (10.6%) | **0.419** | 13 (9.5%) | 67 (7.0%) | 95 (11.5%) | **0.012** |
| Occupation |  |  |  |  |  |  |  |  |  |  |  |  |  |  |  |  |
| Higher occupation | 757 (100.0%) | 819 (100.0%) | 993 (100.0%) | **.** | **.** | **.** | **.** | **.** | 29 (74.4%) | 505 (53.3%) | 743 (62.5%) | **0.001** | 29 (21.6%) | 209 (25.4%) | 183 (25.4%) | **0.524** |
| lower occupation | **.** | **.** | **.** | **.** | 1,643 (100.0%) | 1,208 (100.0%) | 1,113 (100.0%) | **.** | 10 (25.6%) | 443 (46.7%) | 446 (37.5%) | **0.001** | 105 (78.4%) | 614 (74.6%) | 538 (74.6%) | **0.524** |
| Education |  |  |  |  |  |  |  |  |  |  |  |  |  |  |  |  |
| Higher education | 29 (50.0%) | 505 (70.7%) | 743 (80.2%) | **<0.001** | 10 (8.7%) | 443 (41.9%) | 446 (45.3%) | **<0.001** | 44 (100.0%) | 1,054 (100.0%) | 1,296 (100.0%) | **.** | . | . | . | **.** |
| Lower education | 29 (50.0%) | 209 (29.3%) | 183 (19.8%) | **<0001** | 105 (91.3%) | 614 (58.1%) | 538 (54.7%) | **<0.001** | . | . | . | **.** | 138 (100.0%) | 959 (100.0%) | 832 (100.0%) | **.** |
| ≥1 untreated VRF | 224 (33.3%) | 246 (33.7%) | 259 (30.9%) | **0.294** | 610 (40.4%) | 337 (30.2%) | 341 (33.6%) | **<0.001** | 13 (32.5%) | 312 (33.1%) | 347 (31.9%) | **0.587** | 36 (28.1%) | 277 (31.1%) | 255 (32.7%) | **0.280** |
| Hypercholesterolaemia | 56 (12.6%) | 268 (33.4%) | 338 (34.7%) | **<0.001** | 151 (17.6%) | 374 (31.4%) | 497 (45.8%) | **<0.001** | 10 (25.6%) | 355 (34.3%) | 463 (36.2%) | **0.176** | 34 (24.8%) | 295 (31.2%) | 384 (47.2%) | **<0.001** |
| cholesterol-lowering treatment | 33 (68.8%) | 183 (69.6%) | 260 (78.1%) | **0.020** | 93 (67.4%) | 286 (77.1%) | 399 (81.1%) | **0.001** | 9 (90.0%) | 258 (73.3%) | 350 (76.6%) | **0.509** | 26 (78.8%) | 222 (75.5%) | 321 (84.7%) | **0.009** |
| Myocardial infarction | 77 (10.8%) | 73 (9.1%) | 99 (10.2%) | **0.730** | 188 (12.1%) | 98 (8.3%) | 143 (13.1%) | **0.641** | 4 (9.5%) | 81 (8.0%) | 142 (11.1%) | **0.019** | 9 (6.6%) | 83 (8.8%) | 111 (13.6%) | **<0.001** |
| TIA | 78 (11.2%) | 79 (10.0%) | 98 (10.2%) | **0.549** | 230 (15.0%) | 97 (8.1%) | 105 (9.7%) | **<0.001** | 1 (2.5%) | 71 (6.9%) | 128 (10.2%) | **0.002** | 24 (17.8%) | 90 (9.5%) | 82 (10.1%) | **0.144** |
| antithrombotics in TIA or MI | 90 (64.7%) | 100 (71.4%) | 112 (68.3%) | **0.540** | 189 (52.6%) | 134 (73.6%) | 156 (72.2%) | **<0.001** | 2 (40.0%) | 104 (72.2%) | 163 (70.9%) | **0.776** | 25 (78.1%) | 117 (73.1%) | 122 (73.5%) | **0.734** |
| Smoking, current or ex | 414 (58.7%) | 437 (56.0%) | 444 (46.1%) | **<0.001** | 1,045 (66.8%) | 760 (64.8%) | 572 (53.3%) | **<0.001** | 24 (54.5%) | 601 (58.5%) | 572 (45.4%) | **<0.001** | 90 (66.7%) | 586 (62.1%) | 447 (55.6%) | **0.001** |
| pre-stroke BMI>=25 | 77 (43.3%) | 304 (54.9%) | 457 (56.1%) | **0.010** | 197 (53.2%) | 468 (58.9%) | 611 (65.1%) | **<0.001** | 14 (45.2%) | 415 (56.6%) | 621 (58.8%) | **0.168** | 121 (89.0%) | 848 (88.6%) | 706 (84.9%) | **0.017** |
| TOAST |  |  |  |  |  |  |  |  |  |  |  |  |  |  |  |  |
| LAA | 22 (6.1%) | 73 (10.5%) | 77 (8.5%) | **0.453** | 60 (8.6%) | 121 (11.7%) | 102 (10.0%) | **0.465** | 3 (7.1%) | 105 (11.7%) | 106 (9.0%) | **0.109** | 13 (10.6%) | 100 (12.5%) | 73 (9.5%) | **0.166** |
| CE | 85 (23.6%) | 154 (22.2%) | 162 (17.9%) | **0.010** | 167 (24.0%) | 192 (18.6%) | 197 (19.4%) | **0.035** | 8 (19.0%) | 174 (19.5%) | 206 (17.5%) | **0.276** | 21 (17.1%) | 151 (18.8%) | 154 (20.0%) | **0.393** |
| SVO | 74 (20.6%) | 150 (21.6%) | 192 (21.2%) | **0.877** | 204 (29.3%) | 241 (23.4%) | 243 (23.9%) | **0.021** | 8 (19.0%) | 199 (22.3%) | 247 (21.0%) | **0.631** | 38 (30.9%) | 209 (26.1%) | 188 (24.4%) | **0.148** |
| OTH/UND | 88 (24.4%) | 207 (29.8%) | 303 (33.4%) | **0.002** | 157 (22.6%) | 308 (29.8%) | 291 (28.7%) | **0.012** | 15 (35.7%) | 281 (31.4%) | 375 (31.9%) | **0.970** | 37 (30.1%) | 242 (30.2%) | 235 (30.5%) | **0.878** |
| PICH | 56 (15.6%) | 80 (11.5%) | 131 (14.5%) | **0.957** | 77 (11.1%) | 129 (12.5%) | 136 (13.4%) | **0.155** | 5 (11.9%) | 98 (11.0%) | 172 (14.6%) | **0.020** | 9 (7.3%) | 83 (10.3%) | 100 (13.0%) | **0.028** |
| SAH | 35 (9.7%) | 30 (4.3%) | 41 (4.5%) | **0.002** | 31 (4.5%) | 41 (4.0%) | 46 (4.5%) | **0.878** | 3 (7.1%) | 37 (4.1%) | 71 (6.0%) | **0.128** | 5 (4.1%) | 17 (2.1%) | 20 (2.6%) | **0.794** |

Summary statistics are count (%); Percentages refer to those with known value as denominator; when indicated (*) referring to those with relevant VRF diagnosis; p-value for trend across cohorts was calculated using Cochran-Armitage test of trend for categorical variables.

**Abbreviations:** VRFs: Vascular risk factors, BMI: body mass index, BI: Barthel Index, TOAST classification: Trial of Org 10172 in Acute Stroke Treatment classification, LAA: large artery atherosclerosis, CE: cardioembolic, SVO: small vessel occlusion, Oth/UND: other or undefined ischaemic stroke, PICH: primary intracerebral haemorrhage, SAH: subarachnoid haemorrhage ^1^education recorded since 2004, ^2^ “BMI” recorded since 2001, ^3^ “TOAST classification” collected since 1999

# **Table S6: Prevalence ratios of routine/manual occupations and lower education among ethnic minorities vs white individuals**

|  | **Ethniticy** | **aPR** | **95%CI** |
| --- | --- | --- | --- |
| **Occupation** (routine/manual vs non-routine/non-manual) | Black Caribbean | 1.28 | (1.22 - 1.33) |
|  | Black African | 1.07 | (1.00 - 1.15) |
|  | Other | 1.04 | (0.96 - 1.13) |
| **Education** (lower vs higher) | Black Caribbean | 1.10 | (1.01 - 1.19) |
|  | Black African | 0.89 | (0.79 - 0.99) |
|  | Other | 1.04 | (0.93 - 1.18) |

All Poisson regression models additionally adjusted for age, sex, and year of stroke

# **Table S7:** **Adjusted prevalence ratio (95%CI) for pre-stroke VRFs and appropriate primary prevention treatment, mutually adjusted for ethnicity and occupation (model 4) or ethnicity and education (model 5)**

|  | Model | Black Caribbean | Black African | Other | Routine/manual | Lower educational attainment |
| --- | --- | --- | --- | --- | --- | --- |
| Hypertension | Model 4 | 1.28 (1.23-1.33) | 1.46 (1.40-1.53) | 1.20 (1.14-1.27) | 1.08 (1.04-1.12) |  |
|  | Model 5 | 1.28 (1.22-1.33) | 1.49 (1.42-1.56) | 1.24 (1.17-1.32) |  | 1.08 (1.04-1.13) |
| Diabetes | Model 4 | 2.19 (2.01-2.39) | 1.92 (1.72-2.13) | 1.93 (1.72-2.16) | 1.14 (1.05-1.25) |  |
|  | Model 5 | 2.09 (1.90-2.31) | 1.96 (1.75-2.19) | 1.87 (1.64-2.12) |  | 1.22 (1.11-1.34) |
| Hypercholesterol-aemia | Model 4 | 1.13 (1.03-1.23) | 1.09 (0.98-1.20) | 1.20 (1.08-1.34) | 1.14 (1.05-1.23) |  |
|  | Model 5 | 1.12 (1.03-1.23) | 1.13 (1.02-1.25) | 1.19 (1.06-1.33) |  | 0.99 (0.92-1.08) |
| AF | Model 4 | 0.57 (0.48-0.68) | 0.67 (0.54-0.82) | 0.63 (0.50-0.78) | 0.98 (0.88-1.11) |  |
|  | Model 5 | 0.64 (0.53-0.77) | 0.68 (0.54-0.84) | 0.72 (0.57-0.91) |  | 0.81 (0.70-0.93) |
| MI | Model 4 | 0.79 (0.65-0.95) | 0.69 (0.54-0.88) | 1.03 (0.83-1.27) | 1.11 (0.95-1.29) |  |
|  | Model 5 | 0.87 (0.71-1.07) | 0.71 (0.55-0.92) | 1.14 (0.90-1.44) |  | 0.98 (0.82-1.18) |
| TIA | Model 4 | 0.94 (0.79-1.13) | 0.78 (0.61-0.99) | 0.89 (0.69-1.14) | 1.03 (0.89-1.19) |  |
|  | Model 5 | 0.99 (0.80-1.22) | 0.78 (0.59-1.02) | 0.89 (0.66-1.20) |  | 1.03 (0.85-1.24) |
| Smoking | Model 4 | 0.77 (0.73-0.82) | 0.38 (0.34-0.42) | 0.63 (0.58-0.69) | 1.13 (1.08-1.18) |  |
|  | Model 5 | 0.79 (0.74-0.84) | 0.38 (0.34-0.43) | 0.61 (0.55-0.68) |  | 1.11 (1.05-1.16) |
| BMI ≥ 25 | Model 4 | 1.12 (1.04-1.20) | 1.21 (1.14-1.29) | 0.88 (0.79-0.97) | 1.12 (1.06-1.19) |  |
|  | Model 5 | 1.11 (1.03-1.19) | 1.23 (1.15-1.31) | 0.88 (0.79-0.98) |  | 1.12 (1.05-1.18) |
| Antihypertensives treatment* | Model 4 | 1.09 (1.05-1.14) | 1.12 (1.08-1.18) | 1.05 (0.99-1.11) | 1.00 (0.96-1.03) |  |
|  | Model 5 | 1.08 (1.04-1.13) | 1.13 (1.09-1.19) | 1.03 (0.97-1.09) |  | 1.00 (0.96-1.03) |
| Diabetes treatment* | Model 4 | 1.10 (1.04-1.17) | 1.10 (1.02-1.18) | 1.10 (1.03-1.18) | 0.98 (0.93-1.03) |  |
|  | Model 5 | 1.07 (1.00-1.14) | 1.10 (1.01-1.19) | 1.06 (0.97-1.15) |  | 0.99 (0.93-1.05) |
| Cholesterol-lowering treatment* | Model 4 | 0.99 (0.93-1.05) | 0.96 (0.90-1.04) | 1.00 (0.93-1.08) | 1.06 (1.00-1.12) |  |
|  | Model 5 | 0.99 (0.93-1.05) | 0.97 (0.90-1.04) | 1.00 (0.92-1.08) |  | 1.06 (1.01-1.12) |
| Anticoagulants if AF* | Model 4 | 0.90 (0.70-1.14) | 0.93 (0.72-1.20) | 0.76 (0.55-1.50) | 1.01 (0.83-1.23) |  |
|  | Model 5 | 0.90 (0.71-1.15) | 0.91 (0.70-1.18) | 0.75 (0.54-1.03) |  | 1.23 (1.01-1.50) |
| Antithrombotics if TIA or MI* | Model 4 | 0.98 (0.88-1.03) | 0.81 (0.70-0.95) | 0.94 (0.81-1.08) | 0.95 (0.88-1.03) |  |
|  | Model 5 | 0.99 (0.89-1.10) | 0.86 (0.74-1.01) | 0.96 (0.83-1.11) |  | 0.99 (0.90-1.08) |

All Poisson regression models additionally adjusted for age, sex, and year of stroke; reference group for ethnicity: White ethnic group, reference group for occupation: non-routine/non-manual occupation; reference group for education: higher educational attainment; when indicated (*) referring to those with relevant VRF diagnosis; Abbreviations: AF: atrial fibrillation, MI: Myocardial infarction, TIA: transient ischaemic attack, BMI: body mass index

# **Table S8: Adjusted prevalence ratio (95%CI) for pre-stroke VRFs and appropriate primary prevention treatment, including interaction terms between ethnicity and occupation (model 6) or ethnicity and education (model 7)**

|  | Interaction: Ethnicity # Occupation | | | Interaction: Ethnicity # Education | | |
| --- | --- | --- | --- | --- | --- | --- |
| Outcome | **Independent variable** | **aPR (95%CI)** | **p-value** | **Independent variable** | **aPR (95%CI** | **p-value** |
| Hypertension | Black Caribbean | 1.31 (1.20-1.43) | 0.00 | Black Caribbean | 1.31 (1.21-1.43) | 0.00 |
|  | Black African | 1.61 (1.50-1.72) | 0.00 | Black African | 1.61 (1.50-1.73) | 0.00 |
|  | Other | 1.24 (1.12-1.38) | 0.00 | Other | 1.30 (1.17-1.44) | 0.00 |
|  | Routine/manual | 1.10 (1.04-1.16) | 0.00 | Lower education | 1.09 (1.02-1.16) | 0.01 |
|  | Black Caribbean # routine/manual | 0.97 (0.88-1.08) | 0.60 | Black Caribbean # lower education | 0.93 (0.84-1.04) | 0.25 |
|  | Black African # routine/manual | 0.91 (0.83-0.99) | 0.04 | Black African # lower education | 0.97 (0.88-1.07) | 0.55 |
|  | Other # routine/manual | 0.98 (0.86-1.12) | 0.77 | Other # lower education | 0.99 (0.86-1.13) | 0.83 |
| Diabetes | Black Caribbean | 2.24 (1.85-2.71) | 0.00 | Black Caribbean | 1.97 (1.64-2.37) | 0.00 |
|  | Black African | 2.15 (1.80-2.57) | 0.00 | Black African | 2.11 (1.78-2.51) | 0.00 |
|  | Other | 2.14 (1.73-2.65) | 0.00 | Other | 1.95 (1.56-2.44) | 0.00 |
|  | Routine/manual | 1.16 (1.01-1.33) | 0.03 | Lower education | 1.14 (0.96-1.36) | 0.12 |
|  | Black Caribbean # routine/manual | 1.03 (0.83-1.29) | 0.78 | Black Caribbean # lower education | 1.18 (0.93-1.50) | 0.18 |
|  | Black African # routine/manual | 0.90 (0.72-1.13) | 0.37 | Black African # lower education | 1.05 (0.82-1.34) | 0.70 |
|  | Other # routine/manual | 0.94 (0.72-1.23) | 0.66 | Other # lower education | 0.97 (0.71-1.33) | 0.86 |
| Hypercholesterol-  aemia | Black Caribbean | 1.10 (0.91-1.33) | 0.33 | Black Caribbean | 1.05 (0.90-1.22) | 0.51 |
|  | Black African | 1.13 (0.96-1.34) | 0.14 | Black African | 1.17 (1.02-1.34) | 0.03 |
|  | Other | 1.05 (0.84-1.31) | 0.67 | Other | 1.03 (0.84-1.25) | 0.78 |
|  | Routine/manual | 1.13 (1.02-1.26) | 0.02 | Lower education | 0.94 (0.84-1.05) | 0.28 |
|  | Black Caribbean # routine/manual | 0.98 (0.78-1.22) | 0.84 | Black Caribbean # lower education | 1.11 (0.90-1.37) | 0.33 |
|  | Black African # routine/manual | 0.93 (0.74-1.15) | 0.49 | Black African # lower education | 1.02 (0.82-1.27) | 0.85 |
|  | Other # routine/manual | 1.21 (0.92-1.59) | 0.17 | Other # lower education | 1.21 (0.92-1.59) | 0.17 |
| Atrial fibrillation | Black Caribbean | 0.58 (0.39-0.86) | 0.01 | Black Caribbean | 0.74 (0.55-0.99) | 0.05 |
|  | Black African | 0.75 (0.54-1.04) | 0.09 | Black African | 0.69 (0.50-0.96) | 0.03 |
|  | Other | 0.42 (0.25-0.72) | 0.00 | Other | 0.61 (0.39-0.96) | 0.03 |
|  | Routine/manual | 0.99 (0.87-1.13) | 0.88 | Lower education | 0.79 (0.66-0.94) | 0.01 |
|  | Black Caribbean # routine/manual | 1.00 (0.63-1.59) | 0.99 | Black Caribbean # lower education | 0.84 (0.54-1.30) | 0.44 |
|  | Black African # routine/manual | 0.69 (0.42-1.12) | 0.13 | Black African # lower education | 1.00 (0.59-1.70) | 0.99 |
|  | Other # routine/manual | 1.55 (0.80-3.00) | 0.20 | Other # lower education | 1.53 (0.85-2.77) | 0.16 |
| Myocardial infarction | Black Caribbean | 1.04 (0.70-1.53) | 0.86 | Black Caribbean | 0.83 (0.57-1.20) | 0.33 |
|  | Black African | 0.65 (0.43-0.99) | 0.05 | Black African | 0.79 (0.55-1.12) | 0.18 |
|  | Other | 0.98 (0.64-1.51) | 0.94 | Other | 1.04 (0.69-1.58) | 0.85 |
|  | Routine/manual | 1.15 (0.96-1.37) | 0.13 | Lower education | 0.96 (0.76-1.21) | 0.73 |
|  | Black Caribbean # routine/manual | 0.63 (0.40-1.01) | 0.06 | Black Caribbean # lower education | 1.14 (0.69-1.88) | 0.62 |
|  | Black African # routine/manual | 1.07 (0.63-1.82) | 0.81 | Black African # lower education | 0.85 (0.47-1.53) | 0.58 |
|  | Other # routine/manual | 0.98 (0.56-1.70) | 0.93 | Other # lower education | 1.04 (0.56-1.91) | 0.92 |
| TIA | Black Caribbean | 1.01 (0.68-1.50) | 0.96 | Black Caribbean | 0.85 (0.58-1.26) | 0.43 |
|  | Black African | 0.77 (0.52-1.14) | 0.19 | Black African | 0.87 (0.60-1.25) | 0.44 |
|  | Other | 1.05 (0.69-1.59) | 0.83 | Other | 1.08 (0.69-1.68) | 0.74 |
|  | Routine/manual | 1.09 (0.92-1.30) | 0.33 | Lower education | 1.12 (0.88-1.42) | 0.37 |
|  | Black Caribbean # routine/manual | 0.85 (0.54-1.34) | 0.49 | Black Caribbean # lower education | 1.18 (0.71-1.96) | 0.53 |
|  | Black African # routine/manual | 0.93 (0.56-1.56) | 0.79 | Black African # lower education | 0.59 (0.31-1.15) | 0.12 |
|  | Other # routine/manual | 0.60 (0.32-1.09) | 0.09 | Other # lower education | 0.56 (0.27-1.18) | 0.13 |
| Smoking | Black Caribbean | 0.80 (0.71-0.92) | 0.00 | Black Caribbean | 0.82 (0.74-0.91) | 0.00 |
|  | Black African | 0.40 (0.34-0.47) | 0.00 | Black African | 0.37 (0.32-0.44) | 0.00 |
|  | Other | 0.62 (0.53-0.73) | 0.00 | Other | 0.64 (0.55-0.75) | 0.00 |
|  | Routine/manual | 1.12 (1.07-1.18) | 0.00 | Lower education | 1.10 (1.04-1.17) | 0.00 |
|  | Black Caribbean # routine/manual | 0.99 (0.86-1.15) | 0.91 | Black Caribbean # lower education | 0.96 (0.83-1.11) | 0.62 |
|  | Black African # routine/manual | 0.98 (0.79-1.23) | 0.89 | Black African # lower education | 1.07 (0.83-1.38) | 0.59 |
|  | Other # routine/manual | 1.10 (0.90-1.34) | 0.34 | Other # lower education | 0.94 (0.75-1.18) | 0.59 |
| BMI>=25 | Black Caribbean | 1.03 (0.89-1.19) | 0.70 | Black Caribbean | 1.08 (0.97-1.22) | 0.17 |
|  | Black African | 1.21 (1.09-1.35) | 0.00 | Black African | 1.24 (1.13-1.35) | 0.00 |
|  | Other | 0.91 (0.76-1.08) | 0.27 | Other | 0.88 (0.75-1.04) | 0.13 |
|  | Routine/manual | 1.14 (1.05-1.23) | 0.00 | Lower education | 1.13 (1.04-1.23) | 0.01 |
|  | Black Caribbean # routine/manual | 1.05 (0.88-1.24) | 0.59 | Black Caribbean # lower education | 0.95 (0.80-1.11) | 0.50 |
|  | Black African # routine/manual | 0.97 (0.85-1.11) | 0.67 | Black African # lower education | 0.98 (0.85-1.12) | 0.73 |
|  | Other # routine/manual | 0.95 (0.75-1.19) | 0.64 | Other # lower education | 1.01 (0.81-1.27) | 0.92 |
| Hypertension treatment | Black Caribbean | 1.11 (1.02-1.20) | 0.01 | Black Caribbean | 1.13 (1.06-1.21) | 0.00 |
|  | Black African | 1.11 (1.03-1.19) | 0.01 | Black African | 1.18 (1.10-1.26) | 0.00 |
|  | Other | 1.05 (0.96-1.16) | 0.29 | Other | 1.06 (0.97-1.17) | 0.21 |
|  | Routine/manual | 1.00 (0.95-1.06) | 0.88 | Lower education | 1.06 (0.10-1.12) | 0.06 |
|  | Black Caribbean # routine/manual | 0.98 (0.89-1.08) | 0.73 | Black Caribbean # lower education | 0.88 (0.80-0.97) | 0.01 |
|  | Black African # routine/manual | 0.99 (0.91-1.09) | 0.91 | Black African # lower education | 0.89 (0.81-0.97) | 0.01 |
|  | Other # routine/manual | 0.95 (0.84-1.09) | 0.50 | Other # lower education | 0.89 (0.77-1.02) | 0.10 |
| Diabetes treatment | Black Caribbean | 1.06 (0.95-1.18) | 0.33 | Black Caribbean | 1.05 (0.93-1.18) | 0.45 |
|  | Black African | 1.04 (0.94-1.17) | 0.40 | Black African | 1.08 (0.97-1.21) | 0.17 |
|  | Other | 1.03 (0.91-1.17) | 0.65 | Other | 1.07 (0.94-1.22) | 0.33 |
|  | Routine/manual | 0.94 (0.87-1.02) | 0.14 | Lower education | 1.00 (0.90-1.11) | 0.97 |
|  | Black Caribbean # routine/manual | 1.06 (0.93-1.22) | 0.36 | Black Caribbean # lower education | 1.01 (0.86-1.18) | 0.93 |
|  | Black African # routine/manual | 1.06 (0.92-1.22) | 0.41 | Black African # lower education | 0.99 (0.83-1.16) | 0.86 |
|  | Other # routine/manual | 1.15 (0.99-1.34) | 0.07 | Other # lower education | 0.97 (0.80-1.17) | 0.75 |
| Cholesterol lowering treatment | Black Caribbean | 0.98 (0.86-1.13) | 0.81 | Black Caribbean | 0.99 (0.89-1.11) | 0.89 |
|  | Black African | 0.94 (0.83-1.07) | 0.36 | Black African | 0.98 (0.89-1.09) | 0.74 |
|  | Other | 0.96 (0.81-1.13) | 0.61 | Other | 1.04 (0.91-1.19) | 0.53 |
|  | Routine/manual | 1.03 (0.96-1.11) | 0.44 | Lower education | 1.08 (1.01-1.16) | 0.03 |
|  | Black Caribbean # routine/manual | 1.05 (0.90-1.24) | 0.51 | Black Caribbean # lower education | 1.02 (0.88-1.17) | 0.82 |
|  | Black African # routine/manual | 1.04 (0.88-1.22) | 0.68 | Black African # lower education | 0.88 (0.75-1.04) | 0.14 |
|  | Other # routine/manual | 1.13 (0.93-1.37) | 0.23 | Other # lower education | 0.92 (0.76-1.10) | 0.34 |
| Anticoagulants in Atrial fibrillation | Black Caribbean | 1.03 (0.66-1.62) | 0.89 | Black Caribbean | 0.92 (0.62-1.34) | 0.65 |
|  | Black African | 0.95 (0.62-1.45) | 0.80 | Black African | 0.97 (0.63-1.48) | 0.89 |
|  | Other | 0.75 (0.40-1.43) | 0.39 | Other | 0.50 (0.23-1.09) | 0.08 |
|  | Routine/manual | 1.03 (0.82-1.30) | 0.80 | Lower education | 1.17 (0.92-1.48) | 0.20 |
|  | Black Caribbean # routine/manual | 0.83 (0.47-1.48) | 0.54 | Black Caribbean # lower education | 1.04 (0.63-1.74) | 0.87 |
|  | Black African # routine/manual | 1.19 (0.66-2.14) | 0.55 | Black African # lower education | 0.99 (0.57-1.74) | 0.98 |
|  | Other # routine/manual | 0.88 (0.36-2.17) | 0.78 | Other # lower education | 1.36 (0.52-3.58) | 0.53 |
| Antithrombotics in TIA or myocardial infarction | Black Caribbean | 0.92 (0.75-1.13) | 0.44 | Black Caribbean | 0.93 (0.77-1.12) | 0.44 |
|  | Black African | 0.75 (0.57-0.99) | 0.04 | Black African | 0.80 (0.64-0.99) | 0.04 |
|  | Other | 0.92 (0.72-1.18) | 0.53 | Other | 0.86 (0.67-1.10) | 0.23 |
|  | Routine/manual | 0.94 (0.85-1.03) | 0.17 | Lower education | 0.95 (0.86-1.06) | 0.39 |
|  | Black Caribbean # routine/manual | 1.12 (0.87-1.42) | 0.38 | Black Caribbean # lower education | 1.12 (0.88-1.42) | 0.37 |
|  | Black African # routine/manual | 1.02 (0.71-1.47) | 0.92 | Black African # lower education | 1.21 (0.86-1.70) | 0.27 |
|  | Other # routine/manual | 1.00 (0.70-1.41) | 0.99 | Other # lower education | 1.03 (0.70-1.49) | 0.90 |

All Poisson regression models additionally adjusted for age, sex, and year of stroke; Abbreviations: TIA : transient ischaemic attack, BMI: body mass index

# **Table S9A: Risk factor profile, primary prevention treatment and stroke type, stratified by ethnicity**

|  | White | Black Caribbean | Black African | Other | p-value |
| --- | --- | --- | --- | --- | --- |
|  | **N=5,255** | **N=1,303** | **N=1,122** | **N=740** |  |
| Hypertension | 3,084 (61.5%) | 957 (75.5%) | 828 (76.0%) | 473 (66.7%) | **<0.001** |
| antihypertensive treatment* | 2,137 (70.4%) | 741 (78.7%) | 672 (82.0%) | 356 (76.2%) | **<0.001** |
| Diabetes mellitus | 975 (19.3%) | 547 (42.9%) | 378 (34.5%) | 260 (36.1%) | **<0.001** |
| diabetes treatment* | 712 (73.6%) | 434 (79.6%) | 290 (76.7%) | 201 (78.5%) | **0.044** |
| Atrial fibrillation | 1,026 (20.5%) | 128 (10.2%) | 90 (8.3%) | 71 (10.1%) | **<0.001** |
| Newly diagnosed atrial fibrillation | 212 (7.9%) | 39 (4.5%) | 24 (2.8%) | 27 (5.2%) | **<0.001** |
| anticoagulants if AF* | 277 (27.3% | 44 (34.9%) | 40 (44.4%) | 24 (33.8%) | **0.002** |
| Anticoagulants if high-risk AF* | 223 (32.1%) | 39 (37.1%) | 34 (51.5%) | 22 (36.7%) | **0.014** |
| antiplatelets if AF* | 406 (40.4%) | 46 (36.5%) | 28 (31.1%) | 25 (35.2%) | **0.31** |
| Hypercholesterolaemia | 1,222 (29.8%) | 401 (35.6%) | 336 (32.6%) | 237 (36.3%) | **<0.001** |
| cholesterol-lowering treatment* | 924 (76.7%) | 308 (77.4%) | 245 (73.6%) | 182 (77.1%) | **0.61** |
| Myocardial infarction | 621 (12.4%) | 118 (9.5%) | 76 (7.1%) | 82 (11.6%) | **<0.001** |
| TIA | 592 (11.9%) | 128 (10.2%) | 75 (6.9%) | 61 (8.7%) | **<0.001** |
| Antithrombotics if TIA or MI* | 713 (66.0%) | 147 (67.7%) | 76 (56.7%) | 80 (64.5%) | **0.16** |
| Smoking status, current or ex | 3,150 (66.6%) | 620 (52.5%) | 267 (26.2%) | 280 (43.1%) | **<0.001** |
| pre-stroke BMI ≥ 25^2^ | 1,314 (52.6%) | 450 (60.5%) | 515 (69.4%) | 206 (48.2%) | **<0.001** |
| Stroke type |  |  |  |  | **<0.001** |
| Haemorrhagic stroke | 857 (16.8%) | 252 (19.6%) | 264 (23.6%) | 196 (26.6%) |  |
| Ischaemic stroke | 4,248 (83.2%) | 1,037 (80.4%) | 853 (76.4%) | 542 (73.4%) |  |
| TOAST^3^ |  |  |  |  | **<0.001** |
| LAA | 365 (10.2%) | 86 (8.5%) | 67 (7.0%) | 41 (6.8%) |  |
| CE | 898 (25.1%) | 156 (15.4%) | 133 (14.0%) | 101 (16.7%) |  |
| SVO | 666 (18.6%) | 267 (26.3%) | 230 (24.1%) | 119 (19.7%) |  |
| OTH/UND | 1,017 (28.4%) | 309 (30.4%) | 289 (30.3%) | 180 (29.8%) |  |
| PICH | 464 (13.0%) | 146 (14.4%) | 186 (19.5%) | 122 (20.2%) |  |
| SAH | 167 (4.7%) | 51 (5.0%) | 48 (5.0%) | 41 (6.8%) |  |

# **Table S9B: Risk factor profile, primary prevention treatment and stroke type, stratified by occupation and education**

|  | Non-routine/ non-manual | Routine/ manual | p-value | Higher education | Lower education | p-value |
| --- | --- | --- | --- | --- | --- | --- |
|  | **N=2,569** | **N=3,964** |  | **N=2,394** | **N=1,929** |  |
| Hypertension | 1,542 (61.7%) | 2,617 (68.1%) | **<0.001** | 1,450 (61.2%) | 1,356 (71.0%) | **<0.001** |
| antihypertensive treatment* | 1,133 (75.4%) | 1,875 (73.3%) | **0.14** | 1,138 (79.4%) | 1,072 (79.7%) | **0.85** |
| Diabetes mellitus | 583 (23.3%) | 1,055 (27.3%) | **<0.001** | 592 (25.0%) | 585 (30.7%) | **<0.001** |
| diabetes treatment* | 455 (79.4%) | 825 (79.7%) | **0.88** | 451 (77.5%) | 456 (78.6%) | **0.64** |
| Atrial fibrillation | 376 (15.2%) | 576 (15.0%) | **0.89** | 325 (13.9%) | 276 (14.6%) | **0.53** |
| d/c Atrial fibrillation | 94 (5.9%) | 130 (5.9%) | **0.98** | 85 (4.4%) | 96 (6.2%) | **0.023** |
| anticoagulants if AF* | 110 (30.1%) | 144 (25.7%) | **0.14** | 109 (34.0%) | 106 (38.5%) | **0.24** |
| anticoagulants if high-risk AF* | 92 (34.6%) | 114 (30.6%) | **0.28** | 97 (36.5%) | 99 (39.3%) | **0.51** |
| antiplatelets if AF* | 143 (39.2%) | 231 (41.2%) | **0.54** | 122 (38.0%) | 113 (41.1%) | **0.44** |
| Hypercholesterolaemia | 662 (29.8%) | 1,022 (32.6%) | **0.031** | 828 (35.2%) | 713 (37.6%) | **0.11** |
| cholesterol-lowering treatment* | 476 (73.9%) | 778 (77.7%) | **0.076** | 617 (75.3%) | 569 (80.6%) | **0.014** |
| Myocardial infarction | 249 (10.0%) | 429 (11.2%) | **0.13** | 227 (9.7%) | 203 (10.7%) | **0.29** |
| TIA | 255 (10.4%) | 432 (11.4%) | **0.24** | 200 (8.6%) | 196 (10.4%) | **0.049** |
| antithrombotic if TIA or MI* | 302 (68.2%) | 479 (63.3%) | **0.086** | 269 (71.0%) | 264 (73.7%) | **0.4** |
| Smoking, current or ex | 1,295 (52.9%) | 2,377 (62.4%) | **<0.001** | 1,197 (51.4%) | 1,123 (59.7%) | **<0.001** |
| pre-stroke BMI ≥ 25^2^ | 838 (54.2%) | 1,276 (60.7%) | **<0.001** | 1,050 (57.7%) | 865 (60.4%) | **0.11** |
| Stroke type |  |  |  |  |  |  |
| Haemorrhagic stroke | 469 (18.5%) | 632 (16.3%) | **0.025** | 411 (17.2%) | 250 (13.0%) | **<0.001** |
| Ischaemic stroke | 2,073 (81.5%) | 3,248 (83.7%) |  | 1,979 (82.8%) | 1,675 (87.0%) |  |
| TOAST^3^ |  |  | **0.003** |  |  | **<0.001** |
| LAA | 172 (8.8%) | 283 (10.3%) |  | 214 (10.1%) | 186 (11.0%) |  |
| CE | 401 (20.5%) | 556 (20.3%) |  | 388 (18.4%) | 326 (19.2%) |  |
| SVO | 416 (21.2%) | 688 (25.1%) |  | 454 (21.5%) | 435 (25.7%) |  |
| OTH/UND | 598 (30.5%) | 756 (27.6%) |  | 671 (31.8%) | 514 (30.3%) |  |
| PICH | 267 (13.6%) | 342 (12.5%) |  | 275 (13.0%) | 192 (11.3%) |  |
| SAH | 106 (5.4%) | 118 (4.3%) |  | 111 (5.3%) | 42 (2.5%) |  |

Summary statistics are count (%); Percentages refer to those with known value as denominator; when indicated (*) referring to those with relevant VRF diagnosis;

**Abbreviations:** VRFs: Vascular risk factors, BMI: body mass index, TOAST classification: Trial of Org 10172 in Acute Stroke Treatment classification, LAA: large artery atherosclerosis, CE: cardioembolic, SVO: small vessel occlusion, Oth/UND: other or undefined ischaemic stroke, PICH: primary intracerebral haemorrhage, SAH: subarachnoid haemorrhage

# **Table S10: Adjusted prevalence rate ratio (95%CI) of pre-stroke VRFs and primary prevention treatments among ethnic minority groups (model 1) and participants with routine/manual occupation (model 2) and lower educational attainment (model 3), stratified by cohort**

| Outcome | Exposure |  | 1995-2004 | 2005-2014 | 2015-2024 |
| --- | --- | --- | --- | --- | --- |
| Hypertension | **Ethnicity (model 1, vs White)** | **Black Caribbean** | **1.30 (1.21-1.39)** | **1.26 (**1.18-1.34) | **1.30 (**1.22-1.39) |
|  |  | **Black African** | **1.37 (1.23-1.52)** | **1.42 (**1.31-1.54) | **1.54 (**1.45-1.64) |
|  |  | **Other** | **1.06 (0.93-1.21)** | **1.19 (**1.09-1.31) | **1.29 (**1.19-1.40) |
|  | **Occupation (model 2, vs non-routine/non-manual)** | **Routine/manual occupation** | **1.06 (0.99-1.13)** | **1.07 (**1.01-1.14) | **1.15 (**1.08-1.22) |
|  | **Education (model 3, vs higher education)** | **Lower education** | **.** | **1.03 (**0.97-1.10) | **1.09 (**1.03-1.16) |
| Diabetes | **Ethnicity (model 1, vs White)** | **Black Caribbean** | **2.50 (2.12-2.95)** | **2.33 (**1.97-2.75) | **1.95 (**1.72-2.21) |
|  |  | **Black African** | **1.50 (1.09-2.07)** | **1.98 (**1.59-2.46) | **1.88 (**1.65-2.15) |
|  |  | **Other** | **2.24 (1.76-2.85)** | **2.17 (**1.75-2.70) | **1.67 (**1.42-1.96) |
|  | **Occupation (model 2, vs non-routine/non-manual)** | **Routine/manual occupation** | **1.18 (0.99-1.41)** | **1.14 (**0.96-1.35) | **1.31 (**1.16-1.48) |
|  | **Education (model 3, vs higher education)** | **Lower education** | **.** | **1.33 (**1.12-1.58) | **1.14 (**1.00-1.29) |
| Hypercholest-erolaemia | **Ethnicity (model 1, vs White)** | **Black Caribbean** | **1.03 (0.71-1.49)** | **1.04 (**0.89-1.21) | **1.19 (**1.07-1.33) |
|  |  | **Black African** | **0.78 (0.46-1.31)** | **1.09 (**0.91-1.31) | **1.16 (**1.02-1.31) |
|  |  | **Other** | **1.48 (1.01-2.18)** | **1.07 (**0.88-1.30) | **1.23 (**1.07-1.42) |
|  | **Occupation (model 2, vs non-routine/non-manual)** | **Routine/manual occupation** | **1.39 (1.04-1.85)** | **0.95 (**0.84-1.08) | **1.27 (**1.14-1.41) |
|  | **Education (model 3, vs higher education)** | **Lower education** | **.** | **0.91 (**0.80-1.04) | **1.09 (**0.98-1.22) |
| Atrial fibrillation | **Ethnicity (model 1, vs White)** | **Black Caribbean** | **0.45 (0.30-0.67)** | **0.59 (**0.44-0.79) | **0.62 (**0.48-0.80) |
|  |  | **Black African** | **0.51 (0.27-0.97)** | **0.56 (**0.37-0.85) | **0.77 (**0.59-1.00) |
|  |  | **Other** | **0.26 (0.12-0.58)** | **0.61 (**0.42-0.90) | **0.85 (**0.64-1.15) |
|  | **Occupation (model 2, vs non-routine/non-manual)** | **Routine/manual occupation** | **0.97 (0.81-1.17)** | **0.90 (**0.73-1.10) | **0.96 (**0.78-1.19) |
|  | **Education (model 3, vs higher education)** | **Lower education** | **.** | **0.77 (**0.62-0.96) | **0.87 (**0.70-1.08) |
| Myocardial infarction | **Ethnicity (model 1, vs White)** | **Black Caribbean** | **0.57 (0.37-0.88)** | **0.69 (**0.47-1.02) | **1.01 (**0.78-1.30) |
|  |  | **Black African** | **0.57 (0.29-1.12)** | **0.55 (**0.32-0.96) | **0.79 (**0.59-1.07) |
|  |  | **Other** | **0.79 (0.47-1.31)** | **0.93 (**0.59-1.46) | **1.27 (**0.96-1.70) |
|  | **Occupation (model 2, vs non-routine/non-manual)** | **Routine/manual occupation** | **1.05 (0.82-1.35)** | **0.88 (**0.66-1.17) | **1.22 (**0.96-1.55) |
|  | **Education (model 3, vs higher education)** | **Lower education** | **.** | **1.05 (**0.77-1.42) | **0.96 (**0.75-1.22) |
| TIA | **Ethnicity (model 1, vs White)** | **Black Caribbean** | **0.81 (0.58-1.12)** | **0.84 (**0.58-1.22) | **1.15 (**0.87-1.51) |
|  |  | **Black African** | **0.74 (0.44-1.24)** | **0.76 (**0.46-1.27) | **0.81 (**0.58-1.14) |
|  |  | **Other** | **0.79 (0.49-1.28)** | **1.00 (**0.63-1.60) | **0.88 (**0.59-1.30) |
|  | **Occupation (model 2, vs non-routine/non-manual)** | **Routine/manual occupation** | **1.30 (1.02-1.65)** | **0.81 (**0.61-1.07) | **0.91 (**0.70-1.18) |
|  | **Education (model 3, vs higher education)** | **Lower education** | **.** | **1.12 (**0.82-1.54) | **0.79 (**0.60-1.04) |
| Smoking | **Ethnicity (model 1, vs White)** | **Black Caribbean** | **0.79 (0.71-0.86)** | **0.78 (**0.71-0.85) | **0.79 (**0.72-0.88) |
|  |  | **Black African** | **0.37 (0.29-0.48)** | **0.44 (**0.37-0.51) | **0.35 (**0.30-0.41) |
|  |  | **Other** | **0.71 (0.61-0.82)** | **0.53 (**0.45-0.63) | **0.66 (**0.58-0.76) |
|  | **Occupation (model 2, vs non-routine/non-manual)** | **Routine/manual occupation** | **1.09 (1.02-1.17)** | **1.12 (**1.04-1.20) | **1.11 (**1.01-1.21) |
|  | **Education (model 3, vs higher education)** | **Lower education** | **.** | **1.08 (**1.00-1.16) | **1.18 (**1.08-1.29) |
| BMI ≥ 25 | **Ethnicity (model 1, vs White)** | **Black Caribbean** | **1.19 (0.97-1.46)** | **1.16 (**1.05-1.29) | **1.11 (**1.00-1.23) |
|  |  | **Black African** | **1.35 (1.08-1.70)** | **1.16 (**1.03-1.30) | **1.25 (**1.15-1.36) |
|  |  | **Other** | **0.79 (0.52-1.21)** | **0.80 (**0.67-0.97) | **0.92 (**0.81-1.05) |
|  | **Occupation (model 2, vs non-routine/non-manual)** | **Routine/manual occupation** | **1.33 (1.09-1.62)** | **1.08 (**0.98-1.19) | **1.17 (**1.08-1.26) |
|  | **Education (model 3, vs higher education)** | **Lower education** | **.** | **1.06 (**0.96-1.17) | **1.13 (**1.05-1.22) |
| Antihypertensives | **Ethnicity (model 1, vs White)** | **Black Caribbean** | 1.11 (1.01-1.23) | 1.16 (1.09-1.23) | 1.02 (0.97-1.08) |
|  |  | **Black African** | 1.11 (0.97-1.27) | 1.17 (1.08-1.28) | 1.11 (1.05-1.16) |
|  |  | **Other** | 1.00 (0.85-1.18) | 1.09 (0.99-1.20) | 1.02 (0.94-1.09) |
|  | **Occupation (model 2, vs non-routine/non-manual)** | **Routine/manual occupation** | 0.98 (0.90-1.06) | 1.03 (0.97-1.10) | 1.00 (0.96-1.05) |
|  | **Education (model 3, vs higher education)** | **Lower education** | **.** | 1.00 (0.94-1.07) | 0.98 (0.93-1.03) |
| Antidiabetic medication | **Ethnicity (model 1, vs White)** | **Black Caribbean** | 1.16 (1.08-1.24) | 1.09 (1.00-1.19) | 1.07 (0.95-1.19) |
|  |  | **Black African** | 1.02 (0.91-1.14) | 1.05 (0.93-1.19) | 1.15 (1.03-1.28) |
|  |  | **Other** | 1.14 (1.05-1.24) | 1.11 (0.99-1.23) | 1.03 (0.90-1.19) |
|  | **Occupation (model 2, vs non-routine/non-manual)** | **Routine/manual occupation** | 0.94 (0.88-1.01) | 1.04 (0.95-1.13) | 0.99 (0.90-1.09) |
|  | **Education (model 3, vs higher education)** | **Lower education** | **.** | 0.98 (0.90-1.07) | 0.99 (0.90-1.08) |
| Cholesterol-lowering medication | **Ethnicity (model 1, vs White)** | **Black Caribbean** | 1.10 (0.86-1.42) | 0.99 (0.89-1.11) | 1.00 (0.93-1.08) |
|  |  | **Black African** | 0.75 (0.41-1.36) | 0.87 (0.73-1.03) | 1.02 (0.94-1.10) |
|  |  | **Other** | 1.11 (0.83-1.48) | 1.03 (0.90-1.18) | 0.99 (0.89-1.09) |
|  | **Occupation (model 2, vs non-routine/non-manual)** | **Routine/manual occupation** | 1.00 (0.80-1.25) | 1.11 (1.01-1.22) | 1.04 (0.97-1.12) |
|  | **Education (model 3, vs higher education)** | **Lower education** | **.** | 0.99 (0.90-1.09) | 1.08 (1.01-1.16) |
| Anticoagulants if AF | **Ethnicity (model 1, vs White)** | **Black Caribbean** | 1.00 (0.40-2.52) | 1.02 (0.59-1.77) | 0.82 (0.63-1.07) |
|  |  | **Black African** | 1.09 (0.35-3.41) | 0.95 (0.46-1.96) | 0.89 (0.68-1.17) |
|  |  | **Other** | 1.05 (0.24-4.60) | 1.08 (0.53-2.20) | 0.70 (0.49-1.00) |
|  | **Occupation (model 2, vs non-routine/non-manual)** | **Routine/manual occupation** | 0.85 (0.49-1.48) | 0.88 (0.56-1.40) | 1.03 (0.83-1.28) |
|  | **Education (model 3, vs higher education)** | **Lower education** | **.** | 1.36 (0.80-2.33) | 1.07 (0.87-1.31) |
| Antithrombotics if history of TIA or MI | **Ethnicity (model 1, vs White)** | **Black Caribbean** | 0.99 (0.77-1.28) | 1.00 (0.85-1.19) | 0.97 (0.84-1.11) |
|  |  | **Black African** | 0.51 (0.23-1.11) | 0.98 (0.75-1.27) | 0.85 (0.70-1.02) |
|  |  | **Other** | 0.88 (0.60-1.29) | 0.92 (0.73-1.17) | 0.95 (0.79-1.15) |
|  | **Occupation (model 2, vs non-routine/non-manual)** | **Routine/manual occupation** | 0.83 (0.71-0.96) | 1.04 (0.91-1.19) | 1.04 (0.91-1.18) |
|  | **Education (model 3, vs higher education)** | **Lower education** | **.** | 1.00 (0.87-1.14) | 0.99 (0.88-1.13) |

All Poisson regression models additionally adjusted for age, sex, and year of stroke

# **Table S11: Adjusted prevalence ratio (95%CI) for pre-stroke VRFs with interaction terms between ethnicity and cohort (Model 8) or occupation and cohort (Model 9)**

| Vascular risk factor | Ethnicity # Cohort | aPR (95%CI) | p-value | Occupation # Cohort | aPR (95%CI) | p-value |
| --- | --- | --- | --- | --- | --- | --- |
| Hypertension | Black Caribbean | 1.33 (1.24-1.43) | <0.001 | Lower occupation | 1.05 (0.98-1.12) | 0.14 |
|  | Black African | 1.45 (1.32-1.61) | <0.001 |  |  |  |
|  | Other | 1.09 (0.96-1.23) | 0.193 |  |  |  |
|  | 2005-2014 | 1.01 (0.96-1.07) | 0.598 | 2005-2014 | 1.03 (0.95-1.11) | 0.506 |
|  | 2015-2024 | 0.98 (0.93-1.03) | 0.44 | 2015-2024 | 1.02 (0.95-1.10) | 0.603 |
|  | Black Caribbean # 2005-2014 | 0.94 (0.86-1.04) | 0.226 | Lower occupation # 2005-2014 | 1.02 (0.93-1.12) | 0.689 |
|  | Black Caribbean # 2015-2024 | 0.98 (0.89-1.07) | 0.605 | Lower occupation # 2015-2024 | 1.10 (1.00-1.20) | 0.042 |
|  | Black African # 2005-201 | 0.98 (0.86-1.10) | 0.693 |  |  |  |
|  | Black African # 2015-2024 | 1.03 (0.92-1.16) | 0.601 |  |  |  |
|  | Other # 2005-2014 | 1.10 (0.94-1.28) | 0.234 |  |  |  |
|  | Other # 2015-2024 | 1.16 (1.00-1.35) | 0.045 |  |  |  |
| Diabetes | Black Caribbean | 2.56 (2.18-3.01) | <0.001 | Lower occupation | 1.20 (1.01-1.43) | 0.043 |
|  | Black African | 1.53 (1.12-2.07) | 0.007 |  |  |  |
|  | Other | 2.22 (1.75-2.81) | <0.001 |  |  |  |
|  | 2005-2014 | 0.97 (0.84-1.12) | 0.642 | 2005-2014 | 1.12 (0.91-1.37) | 0.273 |
|  | 2015-2024 | 1.50 (1.32-1.72) | <0.001 | 2015-2024 | 1.57 (1.31-1.88) | <0.001 |
|  | Black Caribbean # 2005-2014 | 0.93 (0.74-1.17) | 0.533 | Lower occupation # 2005-2014 | 0.94 (0.73-1.20) | 0.597 |
|  | Black Caribbean # 2015-2024 | 0.76 (0.62-0.94) | 0.01 | Lower occupation # 2015-2024 | 1.09 (0.87-1.35) | 0.457 |
|  | Black African # 2005-201 | 1.39 (0.97-2.00) | 0.076 |  |  |  |
|  | Black African # 2015-2024 | 1.21 (0.87-1.68) | 0.258 |  |  |  |
|  | Other # 2005-2014 | 1.01 (0.74-1.39) | 0.936 |  |  |  |
|  | Other # 2015-2024 | 0.75 (0.56-0.99) | 0.046 |  |  |  |
| Hypercholesterol-aemia | Black Caribbean | 1.12 (0.77-1.62) | 0.548 | Lower occupation | 1.34 (1.01-1.78) | 0.046 |
|  | Black African | 1.09 (0.66-1.79) | 0.733 |  |  |  |
|  | Other | 1.86 (1.27-2.73) | 0.002 |  |  |  |
|  | 2005-2014 | 2.31 (1.96-2.72) | <0.001 | 2005-2014 | 2.66 (2.04-3.46) | <0.001 |
|  | 2015-2024 | 2.90 (2.47-3.40) | <0.001 | 2015-2024 | 2.87 (2.22-3.72) | <0.001 |
|  | Black Caribbean # 2005-2014 | 0.97 (0.65-1.44) | 0.871 | Lower occupation # 2005-2014 | 0.70 (0.51-0.95) | 0.024 |
|  | Black Caribbean # 2015-2024 | 1.06 (0.72-1.56) | 0.768 | Lower occupation # 2015-2024 | 0.96 (0.71-1.30) | 0.774 |
|  | Black African # 2005-201 | 1.12 (0.66-1.89) | 0.669 |  |  |  |
|  | Black African # 2015-2024 | 1.00 (0.60-1.66) | 0.994 |  |  |  |
|  | Other # 2005-2014 | 0.62 (0.40-0.95) | 0.027 |  |  |  |
|  | Other # 2015-2024 | 0.64 (0.42-0.96) | 0.03 |  |  |  |
| Atrial fibrillation | Black Caribbean | 0.44 (0.30-0.66) | <0.001 | Lower occupation | 0.98 (0.81-1.18) | 0.792 |
|  | Black African | 0.50 (0.26-0.93) | 0.03 |  |  |  |
|  | Other | 0.26 (0.12-0.56) | 0.001 |  |  |  |
|  | 2005-2014 | 0.97 (0.86-1.10) | 0.673 | 2005-2014 | 1.00 (0.80-1.24) | 0.994 |
|  | 2015-2024 | 1.07 (0.94-1.22) | 0.309 | 2015-2024 | 0.99 (0.80-1.24) | 0.96 |
|  | Black Caribbean # 2005-2014 | 1.42 (0.87-2.33) | 0.162 | Lower occupation # 2005-2014 | 0.90 (0.68-1.19) | 0.444 |
|  | Black Caribbean # 2015-2024 | 1.38 (0.86-2.22) | 0.179 | Lower occupation # 2015-2024 | 1.02 (0.77-1.35) | 0.911 |
|  | Black African # 2005-201 | 1.32 (0.62-2.79) | 0.476 |  |  |  |
|  | Black African # 2015-2024 | 1.44 (0.73-2.85) | 0.29 |  |  |  |
|  | Other # 2005-2014 | 2.60 (1.09-6.21) | 0.032 |  |  |  |
|  | Other # 2015-2024 | 3.16 (1.37-7.28) | 0.007 |  |  |  |
| Myocardial infarction | Black Caribbean | 0.57 (0.37-0.86) | 0.008 | Lower occupation | 1.06 (0.82-1.35) | 0.666 |
|  | Black African | 0.56 (0.29-1.06) | 0.077 |  |  |  |
|  | Other | 0.76 (0.46-1.26) | 0.288 |  |  |  |
|  | 2005-2014 | 0.79 (0.66-0.94) | 0.01 | 2005-2014 | 0.85 (0.63-1.15) | 0.295 |
|  | 2015-2024 | 1.10 (0.93-1.31) | 0.269 | 2015-2024 | 1.00 (0.76-1.32) | 0.997 |
|  | Black Caribbean # 2005-2014 | 1.25 (0.71-2.20) | 0.447 | Lower occupation # 2005-2014 | 0.85 (0.58-1.24) | 0.397 |
|  | Black Caribbean # 2015-2024 | 1.77 (1.08-2.90) | 0.023 | Lower occupation # 2015-2024 | 1.14 (0.81-1.61) | 0.449 |
|  | Black African # 2005-201 | 1.07 (0.47-2.44) | 0.88 |  |  |  |
|  | Black African # 2015-2024 | 1.37 (0.68-2.75) | 0.381 |  |  |  |
|  | Other # 2005-2014 | 1.26 (0.64-2.47) | 0.503 |  |  |  |
|  | Other # 2015-2024 | 1.63 (0.91-2.92) | 0.097 |  |  |  |
| Transient ischaemic attack | Black Caribbean | 0.84 (0.60-1.17) | 0.297 | Lower occupation | 1.31 (1.03-1.67) | 0.027 |
|  | Black African | 0.80 (0.49-1.32) | 0.388 |  |  |  |
|  | Other | 0.80 (0.50-1.29) | 0.36 |  |  |  |
|  | 2005-2014 | 0.68 (0.57-0.82) | <0.001 | 2005-2014 | 0.92 (0.68-1.23) | 0.568 |
|  | 2015-2024 | 0.79 (0.66-0.95) | 0.014 | 2015-2024 | 0.98 (0.74-1.30) | 0.906 |
|  | Black Caribbean # 2005-2014 | 0.96 (0.59-1.58) | 0.886 | Lower occupation # 2005-2014 | 0.62 (0.43-0.90) | 0.011 |
|  | Black Caribbean # 2015-2024 | 1.36 (0.89-2.09) | 0.158 | Lower occupation # 2015-2024 | 0.71 (0.50-1.01) | 0.055 |
|  | Black African # 2005-201 | 0.85 (0.42-1.73) | 0.661 |  |  |  |
|  | Black African # 2015-2024 | 0.99 (0.55-1.78) | 0.97 |  |  |  |
|  | Other # 2005-2014 | 1.19 (0.61-2.32) | 0.601 |  |  |  |
|  | Other # 2015-2024 | 1.07 (0.58-1.99) | 0.822 |  |  |  |
| Smoking | Black Caribbean | 0.79 (0.72-0.87) | <0.001 | Lower occupation | 1.09 (1.02-1.17) | 0.012 |
|  | Black African | 0.37 (0.29-0.48) | <0.001 |  |  |  |
|  | Other | 0.71 (0.61-0.82) | <0.001 |  |  |  |
|  | 2005-2014 | 1.00 (0.96-1.05) | 0.946 | 2005-2014 | 0.93 (0.85-1.01) | 0.079 |
|  | 2015-2024 | 0.89 (0.85-0.94) | <0.001 | 2015-2024 | 0.76 (0.69-0.83) | <0.001 |
|  | Black Caribbean # 2005-2014 | 0.98 (0.86-1.12) | 0.805 | Lower occupation # 2005-2014 | 1.03 (0.93-1.14) | 0.596 |
|  | Black Caribbean # 2015-2024 | 1.01 (0.88-1.16) | 0.924 | Lower occupation # 2015-2024 | 1.02 (0.92-1.14) | 0.685 |
|  | Black African # 2005-201 | 1.16 (0.87-1.56) | 0.315 |  |  |  |
|  | Black African # 2015-2024 | 0.92 (0.69-1.23) | 0.556 |  |  |  |
|  | Other # 2005-2014 | 0.75 (0.61-0.94) | 0.012 |  |  |  |
|  | Other # 2015-2024 | 0.91 (0.75-1.11) | 0.346 |  |  |  |
| BMI >=25 | Black Caribbean | 1.19 (0.97-1.46) | 0.095 | Lower occupation | 1.26 (1.04-1.53) | 0.017 |
|  | Black African | 1.39 (1.12-1.71) | 0.002 |  |  |  |
|  | Other | 0.80 (0.53-1.21) | 0.298 |  |  |  |
|  | 2005-2014 | 1.16 (1.03-1.30) | 0.015 | 2005-2014 | 1.28 (1.06-1.53) | 0.009 |
|  | 2015-2024 | 1.11 (0.99-1.25) | 0.072 | 2015-2024 | 1.29 (1.08-1.54) | 0.005 |
|  | Black Caribbean # 2005-2014 | 0.97 (0.77-1.22) | 0.811 | Lower occupation # 2005-2014 | 0.85 (0.69-1.06) | 0.15 |
|  | Black Caribbean # 2015-2024 | 0.93 (0.74-1.17) | 0.521 | Lower occupation # 2015-2024 | 0.93 (0.76-1.14) | 0.483 |
|  | Black African # 2005-201 | 0.82 (0.65-1.04) | 0.1 |  |  |  |
|  | Black African # 2015-2024 | 0.91 (0.73-1.14) | 0.411 |  |  |  |
|  | Other # 2005-2014 | 0.99 (0.63-1.56) | 0.96 |  |  |  |
|  | Other # 2015-2024 | 1.16 (0.75-1.79) | 0.508 |  |  |  |

All Poisson regression models additionally adjusted for age, sex, and year of stroke; Abbreviations: BMI: body mass index

# **Table S12: Characteristics of the study population (N=8,515), 1995-2024, stratified by sex and age groups**

|  | male | female | p-value | <65 years | 65-74 years | ≥75 years | p-value |
| --- | --- | --- | --- | --- | --- | --- | --- |
|  | N=4,463 | N=4,052 |  | N=3,136 | N=2,028 | N=3,351 |  |
| Cohort |  |  | **<0.001** |  |  |  | **<0.001** |
| 1995-2004 | 1,448 (32.4%) | 1,459 (36.0%) |  | 866 (27.6%) | 777 (38.3%) | 1,264 (37.7%) |  |
| 2005-2014 | 1,411 (31.6%) | 1,286 (31.7%) |  | 1,003 (32.0%) | 594 (29.3%) | 1,100 (32.8%) |  |
| 2015-2024 | 1,604 (35.9%) | 1,307 (32.3%) |  | 1,267 (40.4%) | 657 (32.4%) | 987 (29.5%) |  |
| Age, years (median, IQR) | 67.1 (57.1-76.9) | 74.8 (62.3-83.6) | **<0.001** | 55.1 (47.4-60.3) | 70.0 (67.5-72.6) | 82.6 (78.8-87.1) | **<0.001** |
| Sex, female |  | 4,052 (100%) |  | 1,183 (37.7%) | 868 (42.8%) | 2,001 (59.7%) | **<0.001** |
| Ethnicity |  |  | **0.012** |  |  |  | **<0.001** |
| White | 2,699 (61.2%) | 2,556 (63.8%) |  | 1,519 (49.0%) | 1,242 (62.0%) | 2,494 (75.1%) |  |
| Black Caribbean | 675 (15.3%) | 628 (15.7%) |  | 488 (15.8%) | 356 (17.8%) | 459 (13.8%) |  |
| Black African | 630 (14.3%) | 492 (12.3%) |  | 729 (23.5%) | 222 (11.1%) | 171 (5.2%) |  |
| Other | 408 (9.2%) | 332 (8.3%) |  | 362 (11.7%) | 182 (9.1%) | 196 (5.9%) |  |
| Occupation |  |  | **<0.001** |  |  |  | **<0.001** |
| non-routine/non-manual | 1,321 (36.4%) | 1,248 (43.0%) |  | 1,077 (42.4%) | 576 (35.8%) | 916 (38.5%) |  |
| routine/manual | 2,308 (63.6%) | 1,656 (57.0%) |  | 1,466 (57.6%) | 1,033 (64.2%) | 1,465 (61.5%) |  |
| Education^1^ |  |  | **0.56** |  |  |  | **<0.001** |
| higher education | 1,354 (55.8%) | 1,040 (54.9%) |  | 1,284 (67.0%) | 514 (52.4%) | 596 (41.8%) |  |
| lower education | 1,074 (44.2%) | 855 (45.1%) |  | 632 (33.0%) | 466 (47.6%) | 831 (58.2%) |  |
| Number of VRFs |  |  | **<0.001** |  |  |  | **<0.001** |
| None | 240 (5.6%) | 359 (9.3%) |  | 336 (11.3%) | 100 (5.1%) | 163 (5.1%) |  |
| 1 or 2 | 2,428 (56.6%) | 2,256 (58.2%) |  | 1,887 (63.2%) | 1,038 (52.8%) | 1,759 (54.8%) |  |
| ≥3 | 1,622 (37.8%) | 1,260 (32.5%) |  | 763 (25.6%) | 829 (42.1%) | 1,290 (40.2%) |  |
| ≥1 untreated VRF | 1,390 (34.3%) | 1,321 (37.6%) | **0.003** | 867 (32.7%) | 635 (34.0%) | **1,209 (39.7%)** | **<0.001** |
| Hypertension | 2,758 (64.6%) | 2,632 (67.6%) | **0.004** | 1,686 (56.1%) | 1,356 (69.5%) | 2,348 (73.1%) | **<0.001** |
| antihypertensive treatment* | 1,956 (72.5%) | 1,950 (76.1%) | **0.003** | 1,157 (70.5%) | 1,022 (77.3%) | 1,727 (75.1%) | **<0.001** |
| Diabetes mellitus | 1,151 (26.8%) | 1,036 (26.3%) | **0.61** | 665 (21.9%) | 681 (34.8%) | 841 (25.8%) | **<0.001** |
| diabetes treatment* | 894 (79.2%) | 743 (73.0%) | **<0.001** | 497 (76.5%) | 542 (81.5%) | 598 (71.9%) | **<0.001** |
| Atrial fibrillation | 610 (14.4%) | 722 (18.6%) | **<0.001** | 199 (6.7%) | 264 (13.7%) | 869 (27.2%) | **<0.001** |
| Newly diagnosed atrial fibrillation | 135 (5.0%) | 167 (7.5%) | **<0.001** | 40 (1.9%) | 79 (6.9%) | 183 (11.3%) | **<0.001** |
| Anticoagulants if AF* | 190 (31.7%) | 195 (27.8%) | **0.12** | 69 (34.7%) | 76 (29.7%) | 240 (28.4%) | **0.21** |
| Anticoagulants if high-risk AF* | 155 (36.8%) | 163 (32.3%) | **0.15** | 43 (43.4%) | 61 (33.3%) | 214 (33.3%) | **0.13** |
| antiplatelets if AF* | 235 (39.2%) | 270 (38.5%) | **0.78** | 64 (32.2%) | 97 (37.9%) | 344 (40.7%) | **0.081** |
| Hypercholesterolaemia | 1,203 (32.5%) | 1,016 (30.8%) | **0.13** | 675 (25.1%) | 628 (38.6%) | 916 (34.2%) | **<0.001** |
| cholesterol-lowering treatment* | 924 (78.6%) | 735 (73.8%) | **0.008** | 456 (69.8%) | 483 (78.7%) | 720 (79.6%) | **<0.001** |
| Myocardial infarction | 542 (12.8%) | 369 (9.5%) | **<0.001** | 209 (7.0%) | 256 (13.2%) | 446 (14.0%) | **<0.001** |
| TIA | 445 (10.6%) | 411 (10.7%) | **0.89** | 225 (7.6%) | 218 (11.4%) | 413 (13.0%) | **<0.001** |
| antithrombotics if TIA or MI* | 570 (65.9%) | 446 (64.6%) | **0.6** | 223 (57.0%) | 271 (65.3%) | 522 (69.7%) | **<0.001** |
| Smoking, current or ex | 2,789 (68.7%) | 1,580 (43.9%) | **<0.001** | 1,699 (59.2%) | 1,187 (63.2%) | 1,483 (50.9%) | **<0.001** |
| pre-stroke BMI ≥ 25^2^ | 1,390 (57.7%) | 1,111 (54.7%) | **0.043** | 1,126 (63.0%) | 638 (60.4%) | 737 (46.2%) | **<0.001** |
| Stroke type |  |  | **0.71** |  |  |  | **<0.001** |
| Haemorrhagic stroke | 842 (19.2%) | 744 (18.9%) |  | 841 (27.1%) | 303 (15.2%) | 442 (13.7%) |  |
| Ischaemic stroke | 3,550 (80.8%) | 3,202 (81.1%) |  | 2,268 (72.9%) | 1,689 (84.8%) | 2,795 (86.3%) |  |
| TOAST^3^ |  |  | **<0.001** |  |  |  | **<0.001** |
| LAA | 324 (9.9%) | 238 (8.1%) |  | 225 (9.2%) | 141 (9.9%) | 196 (8.3%) |  |
| CE | 623 (19.0%) | 683 (23.2%) |  | 283 (11.6%) | 302 (21.2%) | 721 (30.5%) |  |
| SVO | 738 (22.5%) | 564 (19.2%) |  | 532 (21.9%) | 338 (23.7%) | 432 (18.3%) |  |
| OTH/UND | 932 (28.4%) | 879 (29.9%) |  | 715 (29.4%) | 425 (29.8%) | 671 (28.4%) |  |
| PICH | 533 (16.2%) | 393 (13.4%) |  | 452 (18.6%) | 177 (12.4%) | 297 (12.6%) |  |
| SAH | 133 (4.1%) | 181 (6.2%) |  | 227 (9.3%) | 43 (3.0%) | 44 (1.9%) |  |

Summary statistics are count (%); Percentages refer to those with known value as denominator; when indicated (*) referring to those with relevant VRF diagnosis; p-value for trend across cohorts was calculated using Cochran-Armitage test of trend for categorical variables.

**Abbreviations:** VRFs: Vascular risk factors, BMI: body mass index, TOAST classification: Trial of Org 10172 in Acute Stroke Treatment classification, LAA: large artery atherosclerosis, CE: cardioembolic, SVO: small vessel occlusion, Oth/UND: other or undefined ischaemic stroke, PICH: primary intracerebral haemorrhage, SAH: subarachnoid haemorrhage ^1^education recorded since 2004, ^2^ “BMI” recorded since 2001, ^3^ “TOAST classification” collected since 1999
